# Synthesis of Siphonazole B Through Domino Cycloisomerization‐Oxazolonium Ion Rearrangements

**DOI:** 10.1002/chem.202501394

**Published:** 2025-06-27

**Authors:** Filip Paulsen, Sebastian Clementson, Henrik von Wachenfeldt, Michał Antoszczak, Simon Fridolf, Daniel Strand

**Affiliations:** ^1^ Centre for Analysis and Synthesis, Department of Chemistry Lund University Box 124 Lund SE‐221 00 Sweden; ^2^ RG Discovery Medicon Village Lund SE‐223 81 Sweden; ^3^ Department of Medical Chemistry, Faculty of Chemistry Adam Mickiewicz University Uniwersytetu Poznańskiego 8 Poznań 61‒614 Poland

**Keywords:** domino reactions, natural product synthesis, oxidative amidation, rearrangements

## Abstract

4‐Alkenyl‐ and 4‐acyloxazoles represent important substructures across a diverse array of bioactive molecules. Practical methods to synthesize these motifs are, therefore, of considerable interest. Here, we develop a novel domino process wherein a cycloisomerization is followed by a 1,2‐rearrangement via an oxazolonium ion to yield 4‐alkenyloxazoles from an abundant β‐chloro‐*N*‐benzyl propargylamine and acyl chlorides. The synthetic utility of the method is highlighted by its application to both oxazole units of siphonazole B, leading to a concise convergent total synthesis of this natural product.

## Graphical abstract

The study by Strand and co‐workers describes a concise synthesis of siphonazole B. Both oxazole units are formed from a simple propargylamine using a novel domino cycloisomerization‐rearrangement approach. Additional synthetic features include a direct aldol‐type reaction for assembling the tricyclic siphonazole core and an oxidative amidation reaction to install the sensitive (*E*)‐penta‐2,4‐dieneylamide.

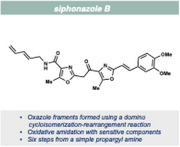

## 1 Introduction

**Scheme 1:**
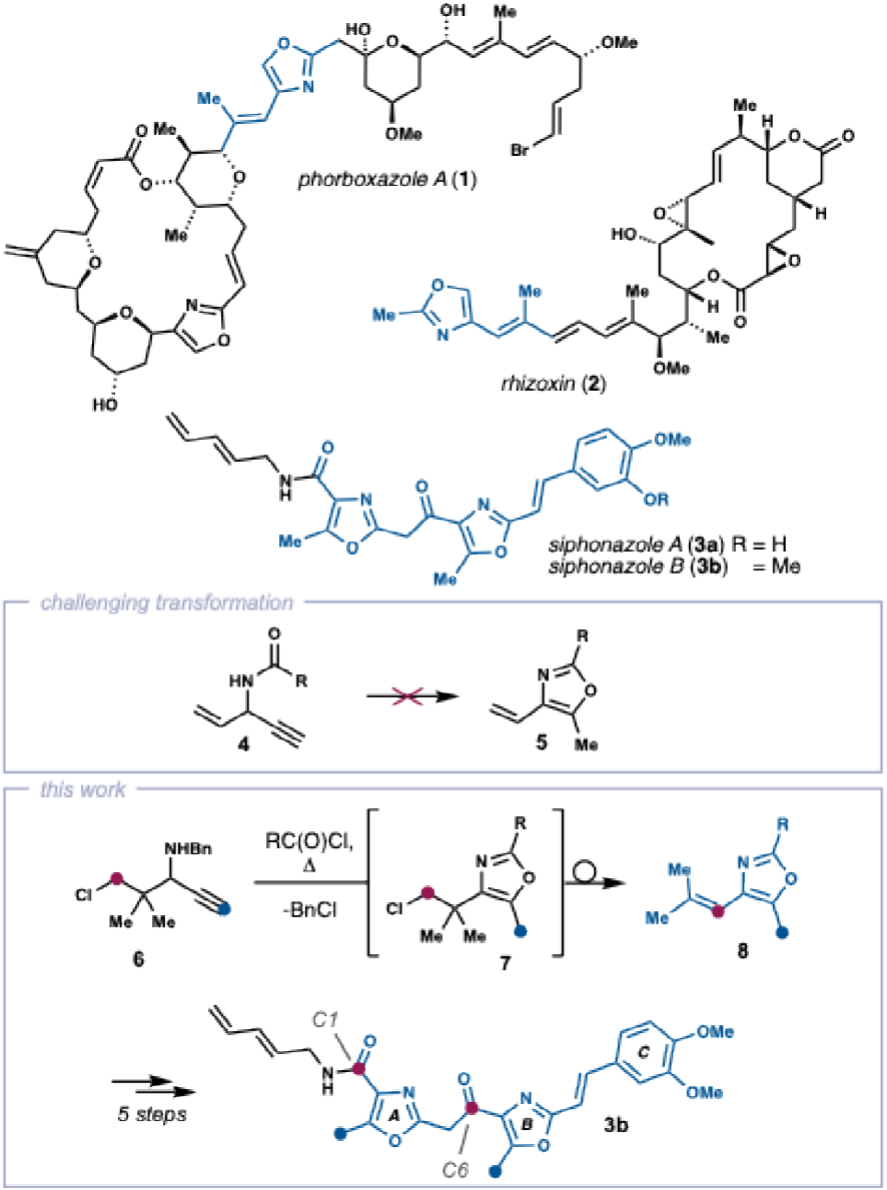
Oxazole natural products and outline of this work. 4‐alkenyl and 4‐acyl oxazole units in natural products highlighted in blue.

4‐Alkenyl and 4‐acyloxazoles are prevalent substructures in biologically active natural products and drugs.^[^ ^1^ ^]^ More complex examples include the highly cytotoxic phorboxazole A (**1**) and rhizoxin (**2**) as well as the antiplasmodial and cytotoxic siphonazoles A and B (**3a**/**b**) (Scheme 1).^[^ ^2^ ^]^ Siphonazoles in particular have received much attention in recent years with total syntheses reported by Moody, Ciufolini, Nikbibn, and Ley, and a formal synthesis by Itami.^[^ ^3^ ^]^ As part of our efforts toward practical syntheses of bioactive oxazoles, we reasoned that the bis‐oxazole motif of siphonazoles could be assembled from 4‐alkenyl oxazole precursors via oxidative cleavage of the alkene to 4‐formyloxazoles. Traditional methods for the synthesis of 4‐alkenyloxazoles include Wittig‐type reactions,^[^ ^4^ ^]^ azirine ring openings,^[^ ^5^ ^]^ and cross‐coupling reactions.^[^ ^6^ ^]^ However, these commonly suffer from lengthy syntheses and cross‐coupling reactions at positions adjacent to aromatic nitrogen atoms are often ineffective.^[^ ^6^, ^7^ ^]^ New step‐economical approaches to this motif therefore remain an important pursuit toward broader applications in synthesis and drug development. A commonly employed tactic for oxazole synthesis is cycloisomerization of *N*‐acyl propargylamines.^[^ ^8^ ^]^ There are, however, no examples of cycloisomerization reactions leading directly to 4‐alkenyloxazole products. In part, this paucity reflects the instability of precursors akin to **4** that carry the requisite olefin side chain. To circumvent this shortcoming, we envisioned a domino process wherein acylation/cycloisomerization of abundant *N*‐benzyl propargylamine **6** would give oxazole **7** ^[^ ^9^ ^]^ with a leaving group at the β‐position; a 1,2‐rearrangement would then lead directly to 4‐alkenyloxazole **8**. Our previous observation that a β‐mesyloxy‐4‐alkyloxazole could be thermally rearranged to a 4‐alkenyloxazole offered critical precedent for this notion.^[^ ^9c^ ^]^

While phenonium ion rearrangements have been of long‐standing synthetic and mechanistic interest, ^[^ ^10^ ^]^ heteroaromatic migrating groups are strikingly underexplored in this context. To our knowledge, only two additional examples of phenonium ion‐type 1,2‐rearrangements with electron‐rich heterocycles have been reported: migration of imidazoles to generate tertiary carbenium ions and a minor side reaction wherein such a rearrangement was observed with an indole. ^[^ ^11^ ^]^

Here, we describe the development of the projected domino process converting *N*‐benzyl propargylamine **6** directly into 4‐alkenyloxazoles by reaction with acyl chlorides. The synthetic utility of this method is highlighted by application to both oxazole units of siphonazole B (**3b**), leading to a concise convergent total synthesis of this natural product.

## 2 Results and Discussion

**Scheme 2:**
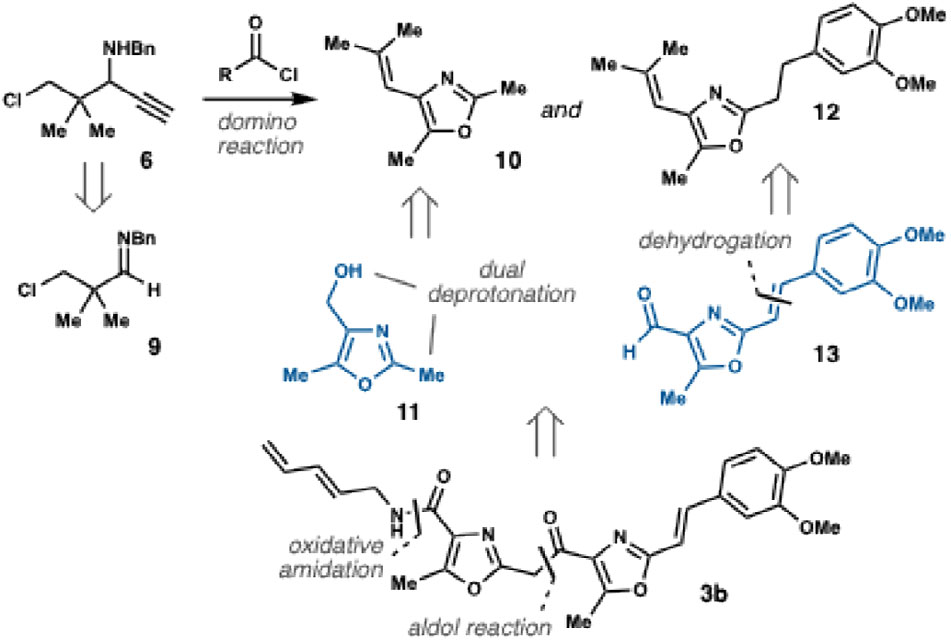
Synthesis plan and key disconnections.

Our initial analysis of siphonazole B (**3b**), outlined in Scheme 2, suggested that its complete tricyclic core could be assembled from 4‐alkenyl oxazoles **10** and **12**; both of which would originate from propargylamine **6** through the projected domino reaction.

To avoid chemoselectivity issues, a saturated two‐carbon linker was chosen for the B/C ring fragment with the intention of introducing the required unsaturation in a subsequent step. Fragments **11** and **13** would then be directly joined in an aldol‐type reaction, leaving only adjustment of the oxidation levels at C1 and C6 to complete the synthesis.

**Scheme 3:**
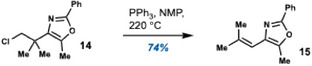
1,2‐Rearrangement of β‐chlorooxazole **14**.

Initially, we sought a practical substrate to evaluate the viability of the projected 1,2‐rearrangement. To this end, β‐chlorooxazole **14** was designed. Pleasingly, this structure efficiently rearranged to 4‐alkenyloxazole **15** when heated to 220 °C in *N*‐methyl pyrrolidone (NMP) (Scheme 3).

When evaluating the influence of additives on the reaction performance, we found that PPh_3_ increased the yield by 10% (Table 1, see also Supporting Information, Table S1). The precise role of the PPh_3_ is not clear, though it seems reasonable to propose that it acts as a nucleophilic catalyst by displacing the chloride, leading to a more efficient rearrangement. Significantly, the successful thermal rearrangement of **14** also translated to the projected domino process. Following a switch of solvent from NMP to MeCN, 4‐alkenyloxazole **15** could be directly obtained in 65% yield from propargylamine **6** and BzCl (Table 1, see also Supporting Information Table S2). The addition of PPh_3_ had a markedly positive effect on both the yield and the reaction time (cf. Entries 1 and 2). Mechanistically, the reaction is thought to proceed via the pathway outlined in Table 1. ^[^ ^9a,c^ ^]^ Initially, an *N*‐acylation reaction releases HCl, which mediates an acid‐catalyzed cyclization to form **18**. The chloride then cleaves the benzyl group to produce exo‐methylene **19**, which isomerizes to oxazole **20**. From **20**, the heterocycle displaces the chloride‐producing oxazolonium ion **21**, which opens to alkene **22** (blue pathway). In line with this pathway, ^1^H NMR spectroscopy of aliquots drawn during the reaction confirms the formation of **22** and its isomerization to 4‐alkenyloxazole **15** over time. We cannot, however, exclude a competing mechanism (red pathway) wherein deprotonation of oxazolonium ion **21**, or its ring‐opened isomer, leads directly to **15**.

**Table 1:** Optimization of the conversion of **6** to **12** and **15**; representative results.^[a]^

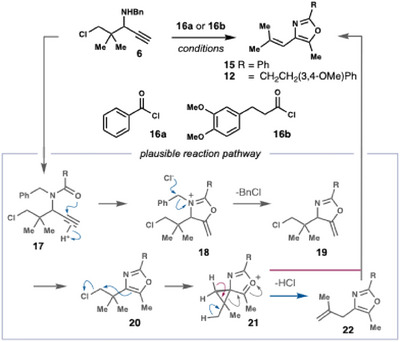					
Entry	Reagent	Solvent	Additive [equiv.]	Time [h]	Yield [%]^[b]^
1	**16a**	MeCN	PPh_3_ (1.05)	1	65 (**15**)
2	**16a**	MeCN	‐	6	26 (**15**)
3	**16b**	MeCN	PPh_3_ (0.4)	1	59 (**12**)
4	**16b**	MeCN	‐	1.5	49 (**12**)
5	**16b**	MeCN	DABCO (0.5)	1	41 (**12**)
6	**16b**	MeCN	P(OMe)_3_	1	43 (**12**)
7	**16b**	MeNO_2_	‐	4	29 (**12**)
8	**16b**	Toluene	‐	1.5	‐

^[a]^ General procedure: Propargylamine **6** and acid chloride (1.1 equiv. entries 1–2 and 1.3 equiv. entries 3–8) were dissolved in the indicated solvent in a sealed vessel and heated to 220 °C.

^[b]^ Measured by ^1^H NMR spectroscopy using DMF as an internal standard. For entries 3–8, a total yield based on the sum of **12** and demethylated side products is given.

**Scheme 4:**
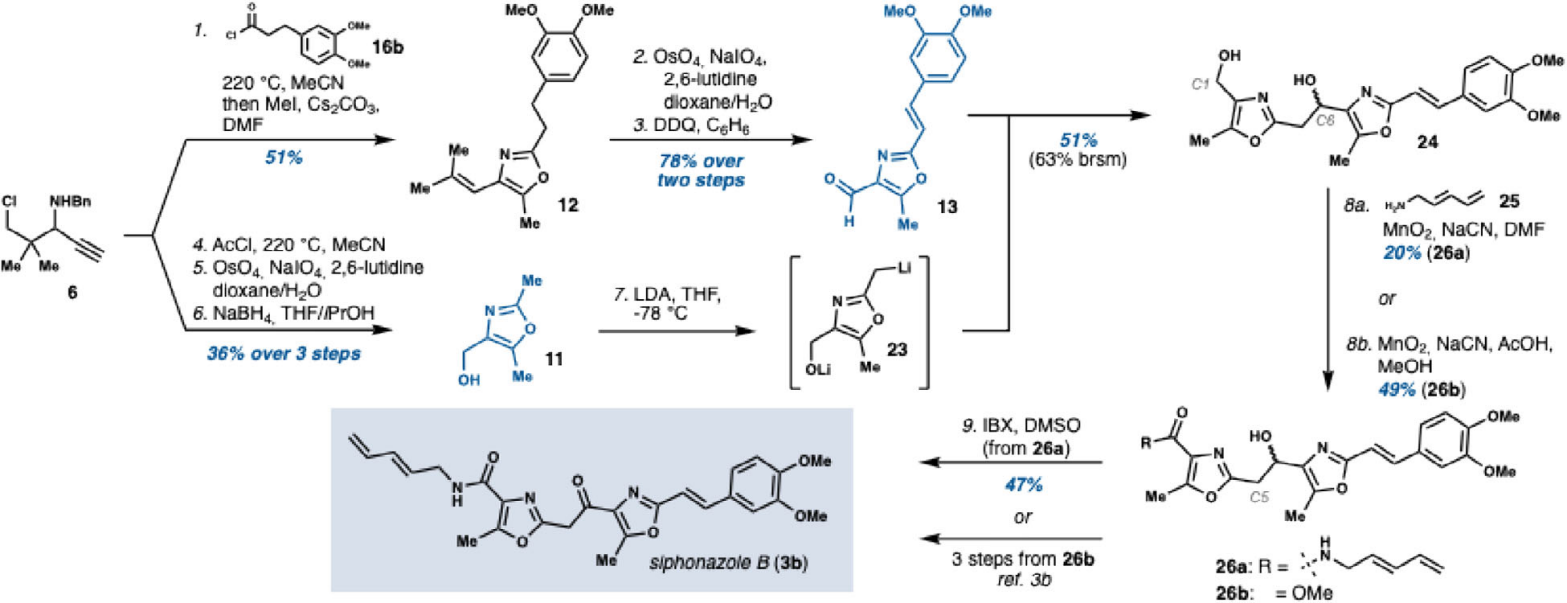
Concise synthesis of siphonazole B (**3b**). BRSM = based on recovered starting material.

With a working procedure in hand, we turned to the syntheses of the projected fragments of siphonazole B (**3b**). Toward the B/C‐ring fragment **13**, we found that acid chloride **16b** readily participated in the reaction with propargylamine **6**. After further refinement of the reaction conditions (Entries 3–8, see also Supporting Information Table S2), up to 59% conversion of the starting material into rearranged products was obtained. By extending the reaction time to 3 hours, the PPh_3_ additive could be omitted, negating the need for a cumbersome workup. Partial cleavage of the methyl ethers occurred during the reaction. This issue was, however, rectified by treatment of the crude reaction mixture with MeI and Cs_2_CO_3_, enabling isolation of oxazole **12** in 51% yield (Scheme 4).

To convert **12** into the complete fragment **13**, the alkenyl unit was first cleaved to the corresponding aldehyde under Lemieux‐Johnson conditions (89% yield).^[^ ^12^ ^]^ Oxidation of the two‐carbon spacer with 2,3‐dichloro‐5,6‐dicyano‐1,4‐benzoquinone (DDQ) then gave aldehyde **13** in 88% yield and > 98:2 stereoselectivity favoring the desired E‐isomer. A similar strategy could also be employed to reach A‐ring fragment **11**. The 4‐alkenyloxazole formed in the domino reaction of propargylamine **6** with AcCl, and the subsequent aldehyde intermediate, are however, both volatile. Therefore, neither was isolated. Instead, the oxidative cleavage and reduction of the crude aldehyde were telescoped to form alcohol **11** in 36% overall yield from **6**.

With straightforward access to **11** and **13**, we turned to the assembly of the tricyclic siphonazole core. An issue previously encountered in the synthesis of siphonazoles is that a basic group such as an ester or amide at the oxazole C4‐position directs metalation to the adjacent (undesired) C5‐position.^[^ ^8b,d,e^ ^]^ Drawing on precedent from Evans' synthesis of phorboxazole A, we reasoned that **11** would allow for a selective lithiation of the more acidic methyl group at the oxazole C2‐position.^[^ ^13^ ^]^ Strategically, this is attractive as it allows for a direct access to the siphonazole framework without relying on transient directing groups as required in prior approaches.^[^ ^3^ ^]^ In practice, double deprotonation of **11** to **23** was accomplished with LDA, and the addition of this intermediate to aldehyde **13** at low temperature produced the sought tricyclic structure **24** in 51% yield, as a single detected regioisomer. The overall efficiency of this step could be further enhanced through the recovery of **11** and **13** (12% and 13% yield, respectively).

At this stage, the remaining hurdle to complete the synthesis was the adjustment of the oxidation levels at C1 and C6. Direct oxidative amidation of **24** is challenging due to the redox sensitivity of both the electron‐rich aromatic rings and the dienyl amine coupling partner **25**. Furthermore, exploratory work showed that the order of oxidation was a critical factor. When the C6 alcohol is oxidized, the resulting ketone tautomerizes to an enol that rapidly adds to any aldehyde present in the reaction mixture. Oxidation of C1 to the carboxylate level thus must be achieved before the C6‐position is reacted, and several protocols for oxidative amidation were explored to this end.^[^ ^14^ ^]^ Gratifyingly, we found that after careful optimization, an excess of MnO_2_ and NaCN as a catalyst gave the desired amide **26a**. Unproductive formation of an imine side‐product from the condensation between amine **25** and the intermediate C1‐aldehyde reflects in a limited yield of 20%. Still, the success of this transformation is noteworthy as it provides the complete siphonazole framework in just five steps from propargylamine **6** (longest linear sequence), leaving only oxidation of the C6‐hydroxyl group to complete the synthesis. This was achieved with 2‐iodoxybenzoic acid (IBX) in DMSO giving siphonazole B (**3b**) in 47% yield along with minor quantities of C5‐hydroxylated byproducts. As an alternative end‐game tactic, we also explored oxidative esterification of **24**. Pleasingly, methyl ester **26b** was obtained in 49% yield using conditions developed for the oxidative amidation, but with methanol in place as the solvent/nucleophile. Formation of **26b** is also noteworthy as it intersects with Moody's synthesis of siphonazole B.^[^ ^3b^ ^]^

## 3 Conclusions

In summary, a novel cycloisomerization‐1,2‐oxazole migration domino reaction was developed that enables the synthesis of 4‐alkenyl oxazoles from an abundant β‐chloro‐*N*‐benzyl propargylamine and aromatic or aliphatic acid chlorides. Applied in a synthesis of siphonazole B (**3b**), the method was used to form both oxazole units and the final structure could be reached in six steps from propargylamine **6**. Additional features of the synthesis include a regioselective aldol‐type reaction to assemble the siphonazole core and chemoselective oxidative amidation/esterification reactions on sensitive substrates. Careful optimization was critical to several steps in the synthesis and highlights the need for further advances, particularly in the development of late‐stage oxidation reactions. Studies in this direction and investigations of the scope of 1,2‐rearrangements of heterocycles via phenonium ion‐type intermediates are underway and will be reported in due course.

## Supporting Information

Synthetic procedures and spectroscopic data for all new compounds. Summary of crystallographic information for S4.^[^ ^15^ ^]^ The authors have cited additional references within the Supporting Information.^[^ ^16^, ^17^, ^18^, ^19^ ^]^

## Conflict of Interest

The authors declare no conflict of interest.

## Supporting information



Supporting Information

## Data Availability

The data that support the findings of this study are available in the supplementary material of this article.
