# Supplementary material for: Synthesis of Siphonazole B Through Domino Cycloisomerization‐Oxazolonium Ion Rearrangements
Source: Chemistry. 2025 Jun 27;31(40):e202501394. doi: 10.1002/chem.202501394 (PMC12271995; doi:10.1002/chem.202501394)

Supporting Information

Synthesis of Siphonazole B Through Domino Cycloisomerization-Oxazolonium Ion Rearrangements

Filip Paulsen,[a,b] Sebastian Clementson,[a] Henrik von Wachenfeldt,[a,b] Michał Antoszczak,[c] Simon Fridolf[a] and Daniel Strand*[a]

a) Centre for Analysis and Synthesis, Department of Chemistry, Lund University Box 124, SE-221 00 Lund, Sweden; b) RG Discovery, Medicon Village SE-223 81 Lund, Sweden.; c) Department of Medical Chemistry, Faculty of Chemistry, Adam Mickiewicz University Uniwer-sytetu Poznańskiego 8, 61–614, Poznań, Poland.

E-mail: daniel.strand@chem.lu.se

Index

I. General procedures. *S2*

II. Experimental procedures and characterization data for compounds
S2**, **9**, **6**, **14/15**, **16b**, **12**, **S4**, **13**, **11**, **24**, **S6**, **25**, **26a/b**, and **3b *S4*

III. Optimization of the oxazole-rearrangement *S20*

IV. Optimization of the domino oxazole formation-rearrangement *S21*

*V. Crystal structure data for compound **S4*** *S22*

*VI. NMR spectra for compounds **S2**, **9**, **6**, **14/15**, **16b**, **12**, **S4**, **13**, **11**, **24**, **S6**, **25**, **26a/b**, and **3b*** *S23*

SAFETY STATEMENT: No unusual or unexpected safety issues were encountered in this work. Sealed vessels used at elevated temperatures must be designed to withstand pressure. These vessels were inspected for damages prior to each reaction.

I. General procedures

Synthesis, general: All reactions were carried out in oven-dried glassware under a nitrogen atmosphere unless otherwise stated. Reactions were heated using a metal block on a hotplate with feedback temperature control, unless otherwise stated. Room temperature (RT) refers to temperatures in a range between 20 – 25 °C. Microwave heated reactions were performed in closed vessels using a Biotage Initiator$^+$ (Fourth Generation Microwave Synthesizer) with external temperature control. Mixtures were concentrated under reduced pressure by rotary evaporation. Reactions were monitored by TLC using aluminum-backed plates (Merck 60F$_{254}$ silica gel) and visualized with UV-light (254 nm) and/or staining with alkaline $KMnO_4$,

phosphomolybdic acid stain, anisaldehyde stain, or DDQ (10% in toluene). Preparative chromatography was performed using silica gel (Acros 40 – 60 µm, 60 Å) on a flash-purification system (Biotage Isolera One). Petroleum ether refers to the fraction boiling at 40 – 60 °C. For known compounds (references given) synthesized through new or modified procedures, copies of ^{1}H NMR spectra are provided. For the previously known late-stage methyl ester **26b**, and for siphonazole B (**3b**), ^{1}H and ^{13}C{^{1}H} spectra are provided.

Reagents and solvents: Anhydrous solvents (THF, CH_2Cl_2, Et_2O, toluene) were freshly collected after filtration through alumina-columns on a solvent-purification system. Anhydrous DMSO was obtained as extra dry and used as received. Acetonitrile and 1,2-dichloroethane (DCE) were freshly distilled from CaH_2. Benzene was distilled from sodium/benzophenone ketyl and stored over molecular sieves (4Å). Dioxane was stored over molecular sieves (4Å) and passed through a column of basic alumina prior to use. LDA was prepared by a dropwise addition of *n*-BuLi (1.60 mL, 1.6 M in hexanes, 2.56 mmol) to a stirred solution of diisopropylamine (395 µl, 2.82 mmol) in THF (2.0 mL) at -78 °C. The resulting solution was warmed to 0 °C, over 5 min, and then cooled to -78 °C. All other solvents and reagents were bought from commercial suppliers and used as received.

NMR spectroscopy: NMR spectra were recorded on a Bruker Ultrashield 400 plus (^{1}H at 400 MHz and ^{13}C{^{1}H} at 101 MHz) or on a Bruker Avance II (^{1}H at 500 MHz and ^{13}C{^{1}H} at 126 MHz) and processed using MestReNova version 12.2.4. Chemical shifts are given in ppm downfield from $SiMe_4$ using the residual peak of the solvent as reference: $CDCl_3$: δ = 7.26 for ^{1}H and δ = 77.2 for ^{13}C{^{1}H}; $(CD_3)_2CO$: δ = 2.84 for ^{1}H and δ = 206.26 for ^{13}C{^{1}H}); C_6D_6 : δ = 7.16 for ^{1}H. ^{1}H NMR spectra are reported as follows: chemical shifts (δ, ppm), multiplicity (s = singlet, d = doublet, t = triplet, q = quartet, sept. = septet, dd = doublet of doublets, dt = doublet of triplets, m = multiplet, br. = broad, app. = apparent), coupling constant (Hz) and integration. ^{13}C{^{1}H} NMR spectra are reported in chemical shifts. References are given for

known compounds. For quantitative ^{1}H NMR spectroscopy measurements, a relaxation delay of 20 s was used.

Mass spectrometry: Mass spectra (HRMS) were recorded on a Micromass Q-TOF spectrometer (ESI) or on a LTQ Velos PRO orbitrap mass spectrometer (Thermo Scientific) (ESI).

IR spectroscopy: IR spectra were recorded on a Bruker ALPHA-P in ATR mode and selected peaks are reported as follows: wavenumbers (cm^{-1}), description (w = weak, m = medium, s = strong, br. = broad).

*II. Experimental procedures and characterization data for compounds **S2**, **9**, **6**, **14/15**, **16b**, **12**, **S4**, **13**, **11**, **24**, **S6**, **25**, **26a/b**, and **3b***

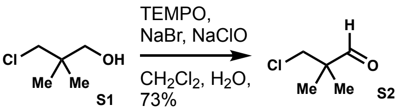

3-Chloro-2,2-dimethylpropanal (S2).[1] To a solution of 2,2-dimethyl-3-chloro-propanol (12.0 g, 97.9 mmol,) and TEMPO (765 mg, 4.89 mmol), in CH_2Cl_2 (65 mL), was added a solution of NaBr (1.01 g, 9.82 mmol) dissolved in water (5.0 mL). The resulting orange mixture was stirred in air and cooled with an external ice/water bath. A solution of sodium hypochlorite (60 mL, 13% available chlorine, 1.8 – 2.2 M) and $NaHCO_3$ (3.29 g, 39 mmol) in water (48 mL), was then added over one hour using a dropping funnel. During addition, the internal temperature of the reaction mixture was kept below 10 °C (internal thermometer control). Shortly after the start of addition, the reaction mixture turned dark brown. Upon complete addition of the hypochlorite solution, TLC indicated full consumption of the alcohol. The reaction was quenched by addition of $Na_2S_2O_3$ (30% aq.) until the brown color faded. The phases were then separated and the water phase was extracted with CH_2Cl_2 (4 x 40 mL). The combined organic

[1] Known compound: F. Effenberger; J. Eichhorn; J. Roos *Tetrahedron: Asym.* **1995**, *6*, 271.

phases were washed with brine, dried using a phase-separator, and carefully concentrated under reduced pressure. The remaining orange liquid was distilled under reduced pressure (56 °C, 47 mbar) to give aldehyde **S2** (8.56 g, 73%) as a colorless liquid, >95% by NMR spectroscopy and a single spot by TLC. ^{1}H and ^{13}C{^{1}H} NMR spectroscopy data were in agreement with those previously reported.

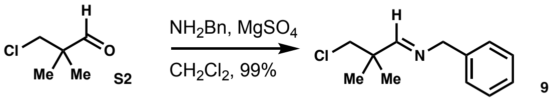

***N*-(3-Chloro-2,2-dimethylpropylidene)-1-phenylmethanamine (9).**[2] To a stirred suspension of aldehyde **S2** (1.00 g, 8.29 mmol) and anhydrous $MgSO_4$ (2.00 g) in CH_2Cl_2 (15 mL) was added benzylamine (0.86 mL, 7.87 mmol) in one portion. The reaction mixture was stirred for 16 h, then filtered and concentrated under reduced pressure to give imine **9** (1.64 g, 99%) as a colorless liquid, >95% by NMR spectroscopy and a single spot by TLC. ^{1}H and ^{13}C{^{1}H} NMR spectroscopy data were in agreement with those previously reported.

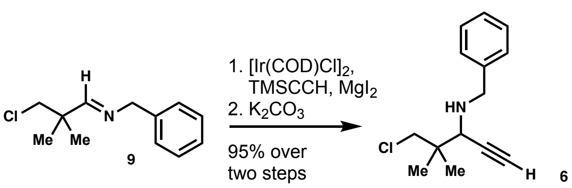

***N*-Benzyl-5-chloro-4,4-dimethylpent-1-yn-3-amine (6).** To a stirred suspension of magnesium iodide (344 mg, 1.23 mmol) and bis(1,5-cyclooctadiene)diiridium dichloride (277 mg, 0.413 mmol) in THF (53 mL) was added imine **9** (8.65 g, 41.2 mmol), followed by ethynyltrimethylsilane (9.0 mL, 62 mmol), in one portion respectively. A color change from bright red to dark brown was observed within 10 min after addition. After stirring for 20 h, a 93 : 7 ratio of product to starting material was observed by ^{1}H NMR spectroscopy and a second portion of bis(1,5-cyclooctadiene)diiridium dichloride (71 mg, 0.106 mmol) was added. The

[2] Known compound: P. Sulmon; N. De Kimpe; R. Verhé; L. De Buyck; N. Schamp *Synthesis* **1986**, *1986*, 192.

mixture was stirred for a further 6 h at which point ^{1}H NMR spectroscopy indicated a 98 : 2 ratio. Methanol (2.0 mL) was then added, and the volatiles were evaporated under reduced pressure. The resulting crude was dissolved in anhydrous methanol (170 mL) and K_2CO_3 (5.67 g, 41 mmol) was added. The suspension was stirred for 2.5 h at room temperature at which time TLC indicated complete consumption of the TMS-protected propargylic amine intermediate. The mixture was concentrated under reduced pressure, re-dissolved in CH_2Cl_2 (150 mL), and washed with brine (200 mL). The aqueous phase was re-extracted with CH_2Cl_2 (2 x 30 mL), and the combined organic phases were washed with brine (50 mL), dried ($MgSO_4$), filtered through a small plug of celite and concentrated under reduced pressure. Purification by silica gel column chromatography (0 – 5% EtOAc/heptane) gave propargylic amine **6**.

Yield: 9.24 g, 95%. Obtained as a brown oil, >95% by pure NMR spectroscopy and a single spot by TLC.

R*f*: 0.41 (5% EtOAc/petroleum ether). Stains yellow with $KMnO_4$ stain.

^{1}H NMR (400 MHz, CDCl$_3$): δ 7.40 – 7.36 (m, 2H), 7.36 – 7.30 (m, 2H), 7.28 – 7.23 (m, 1H), 4.08 (d, *J* = 12.8 Hz, 1H), 3.77 (d, *J* = 12.8 Hz, 1H), 3.71 (d, *J* = 10.5 Hz, 1H), 3.52 (d, *J* = 10.5 Hz, 1H), 3.32 (d, *J* = 2.2 Hz, 1H), 2.35 (d, *J* = 2.2 Hz, 1H), 1.22 (br. s, 1H), 1.09 (s, 3H), 1.06 (s, 3H) ppm.

^{13}C{^{1}H} NMR (100 MHz, CDCl$_3$): δ 140.1, 128.6, 128.4, 127.2, 83.6, 73.0, 55.5, 53.6, 52.6, 39.6, 21.7, 21.1 ppm.

FTIR (ATR): 3295 (br. m), 3029 (m), 1454 (s), 1385 (m), 1366 (m) cm^{-1}.

HRMS-ESI (*m/z*): $[M + H]^+$ Calcd for $C_{14}H_{19}NCl$ 236.1206; Found 236.1187.

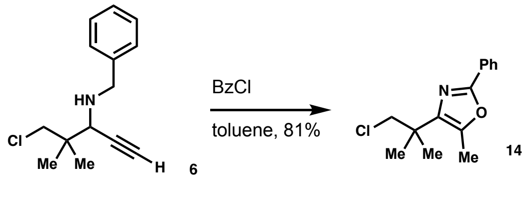

4-(1-Chloro-2-methylpropan-2-yl)-5-methyl-2-phenyloxazole (14). To a solution of benzoyl chloride **16a** (0.32 mL, 2.76 mmol) in toluene (1.0 mL) was added propargyl amine **6** (500 mg, 2.12 mmol). The resulting solution was heated to 150 °C using microwave irradiation. After 0.5 h, TLC indicated full conversion of propargyl amine **9**. The solution was concentrated under reduced pressure. The crude product was purified by silica gel flash column chromatography (0 – 25% EtOAc/heptane) to give chloride **14**.

Yield: 81%, 430 mg. Obtained as a brown oil, >95% pure by NMR and a single spot by TLC.

R*f*: 0.26 (5% EtOAc/heptane).

^{1}H NMR (400 MHz, $CDCl_3$): δ 7.99 – 7.96 (m, 2H), 7.45 – 7.38 (m, 3H), 3.76 (s, 2H), 2.47 (s, 3H), 1.45 (s, 6H) ppm.

^{13}C{^{1}H} NMR (100 MHz, $CDCl_3$): δ 158.4, 143.6, 139.7, 129.8, 128.7, 128.0, 126.1, 55.5, 37.4, 25.6, 12.5 ppm.

FTIR (ATR): 2972 (br, w), 1613 (w), 1559 (m), 1448 (m), 1259 (w) cm^{-1}.

HRMS-ESI (*m/z*): $[M + H]^+$ Calcd for $C_{14}H_{17}ClNO$ 250.0999; Found 250.1000.

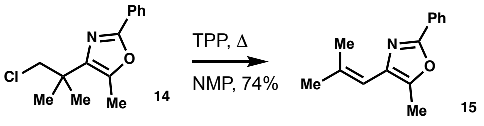

5-Methyl-4-(2-methylprop-1-en-1-yl)-2-phenyloxazole (15). To a solution of chloride **14** (50 mg, 0.2 mmol) in *N*-methyl pyrrolidine (NMP) (0.25 mL) was added PPh_3 (10.5 mg, 0.04 mmol). The resulting solution was heated to 220 °C using microwave irradiation. After 3 h, ^{1}H NMR spectroscopy of an aliquot indicated full conversion of chloride **14**. The solution was directly evaporated onto silica and purified by silica gel flash column chromatography (0 – 25% EtOAc/heptane) to give oxazole **15**.

Yield: 74% by ^{1}H NMR spectroscopy of the crude reaction mixture using mesitylene as an internal standard. Isolated yield: 59%, 25 mg. Obtained as a brown oil, >95% pure by NMR and a single spot by TLC.

R*f*: 0.42 (10% EtOAc/heptane).

^{1}H NMR (400 MHz, CDCl$_3$): δ 8.05 – 7.99 (m, 2H), 7.46 – 7.38 (m, 3H), 5.95 (s, 1H), 2.34 (s, 3H), 2.07 (s, 3H), 1.92 (d, *J* = 1.4 Hz, 3H) ppm.

^{13}C{^{1}H} NMR (100 MHz, CDCl$_3$): δ 159.0, 144.5, 137.9, 134.7, 129.8, 128.7, 128.0, 126.1, 113.6, 26.9, 19.8, 10.9 ppm.

FTIR (ATR): 3295 (br. w), 2979 (w), 1679 (br. s), 1449 (m), 1241 (br. s) cm^{-1}.

HRMS-ESI (*m*/*z*): [M + H]$^+$ Calcd for $C_{14}H_{16}NO$ 214.1232; Found 214.1230.

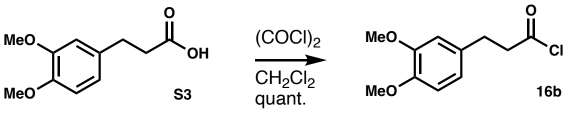

3-(3,4-Dimethoxyphenyl)propanoyl chloride (16b). To a stirred solution of 3-(3,4-dimethoxyphenyl)-propanoic acid (**S3**) (2.00 g, 9.51 mmol) in anhydrous CH_2Cl_2 (60 mL), at 0 °C, was added oxalyl chloride (0.97 mL, 11.5 mmol) dropwise. The solution immediately turned bright yellow. The reaction mixture was stirred at room temperature for 16 h, during which time the color faded to pale yellow. Upon consumption of the acid (TLC control; small samples of the reaction mixture were drawn and quenched with anhydrous methanol), the reaction mixture was concentrated under reduced pressure. The obtained solids were melted by gentle heating under reduced pressure (1 mbar) to give acid chloride **16b**. The material was used in the subsequent step without further purification.

Yield: 2.17 g (quant.). Obtained as an off-white solid, >95% by NMR spectroscopy and a single spot by TLC.

R$_f$: (methyl ester) 0.42 (33% EtOAc/petroleum ether). Stains yellow with $KMnO_4$ stain.

^{1}H NMR (400 MHz, $CDCl_3$): δ 6.81 (d, *J* = 8.1 Hz, 1H), 6.73 (dd, *J* = 8.1 Hz, 2.0 Hz), 6.71 (d, *J* = 2.0 Hz, 1H), 3.88 (s, 3H), 3.86 (s, 3H), 3.20 (t, *J* = 7.5 Hz, 2H), 2.96 (t, *J* = 7.5 Hz, 2H) ppm.

^{13}C{^{1}H} NMR (100 MHz, $CDCl_3$): δ 173.3, 149.2, 148.0, 131.3, 120.4, 111.7, 111.5, 56.1, 56.0, 49.0, 30.8.

FTIR (ATR): 3000 (w), 2936 (w), 2892 (w), 1783 (s), 1609 (w), 1591 (m), 1514 (s) cm^{-1}.

HRMS: Satisfactory HRMS data were not obtained due to the instability of the product. The identity of **16b** was inferred by its successful participation in a subsequent reaction with methanol forming its known methyl ester.

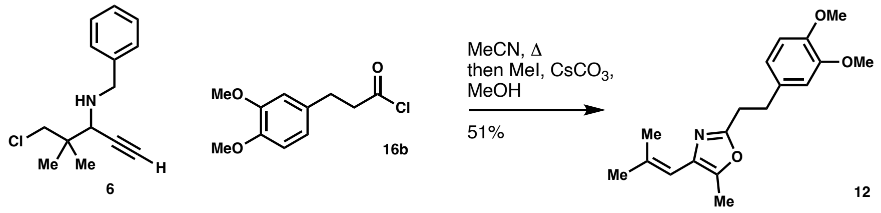

2-(3,4-Dimethoxyphenethyl)-5-methyl-4-(2-methylprop-1-en-1-yl)oxazole (12). [3] To a solution of acyl chloride **16b** (529 mg, 2.31 mmol) in acetonitrile (2.0 mL) was added propargyl amine **6** (519 mg, 2.20 mmol). The resulting solution was heated to 220 °C using microwave irradiation. After 2.5 h, ^{1}H NMR spectroscopy of an aliquot indicated 75% conversion from the intermediate chloro-oxazole. The mixture was then heated for an additional 30 min at 220 °C to give full consumption of the intermediate. The solution was concentrated under reduced pressure at 90 °C until no benzyl chloride could be detected by ^{1}H NMR. To the resulting oil was added cesium carbonate (1436 mg, 4.41 mmol) and DMF (8.0 mL) followed by methyl iodide (0.49 mL, 4.4 mmol) and the mixture was stirred at RT for 4 h. Upon complete

[3] Omission of triphenylphosphine compared to the optimized protocol simplified the workup and had only a minor impact on the yield of the reaction.

consumption of the phenol by-products (TLC control) the mixture was diluted with ethyl acetate (80 mL) and washed with brine (80 mL) containing ammonia (2.0 mL, sat. aq.) followed by brine (2.0 x 80 mL). The organic phase was dried using a phase separator and concentrated under reduced pressure. The crude product was purified by silica gel flash column chromatography (0 – 25% EtOAc/heptane) to give alkene **12**.

Yield: 336 mg (51%). Obtained as a pale-yellow oil, >95 % pure by NMR spectroscopy and a single spot by TLC.

R*f*: 0.50 (20% EtOAc/petroleum ether). Stains yellow with $KMnO_4$ stain.

^{1}H NMR (400 MHz, $CDCl_3$): δ 6.83 – 6.71 (m, 3H), 5.86 (app. s, 1H), 3.85 (s, 3H), 3.84 (s, 3H), 3.08 – 2.93 (m, 4H), 2.22 (s, 3H), 1.98 (d, *J* = 0.8 Hz, 3H), 1.87 (d, *J* = 0.8 Hz, 3H) ppm.

^{13}C{^{1}H} NMR (100 MHz, $CDCl_3$): δ 161.3, 148.9, 147.6, 143.7, 137.1, 133.5, 133.0, 120.3, 113.7, 111.8, 111.4, 56.0, 55.9, 33.0, 30.5, 26.8, 19.8, 10.7 ppm.

FTIR (ATR): 2961 (m), 2916 (m), 2834 (w), 1584 (m), 1514 (s), 1450 (m), 1418 (m) cm^{-1}.

HRMS-ESI (*m/z*): $[M + H]^+$ Calcd for $C_{18}H_{24}NO_3$ 302.1756; Found 302.1750.

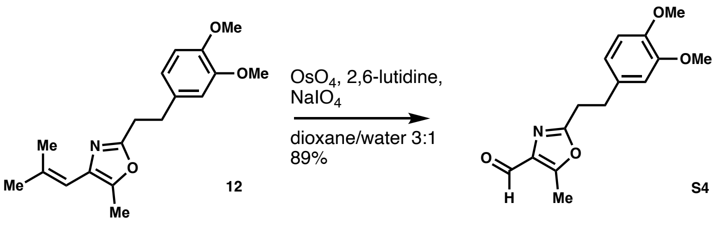

2-(3,4-Dimethoxyphenethyl)-5-methyloxazole-4-carbaldehyde (S4). To a stirred solution of alkenyl-oxazole **12** (1.52 g, 5.04 mmol) in dioxane:water (3:1, 51 mL) was added 2,6-lutidine (1.2 mL, 10 mmol) followed by OsO_4 (1.0 mL, 2.5% in *t*-BuOH, 0.10 mmol) in one portion respectively. The resulting pale orange solution was stirred for 5 min at RT and then sodium periodate (4.32 g, 20.2 mmol) was added in one portion. The resulting suspension was stirred for a further 2 h at which point TLC indicated complete consumption of the starting material

and the reaction was quenched by the addition of $Na_2S_2O_3$ (16 mL, 300 g/l, aq.). After stirring for 30 min, the mixture was poured on brine (150 mL) and extracted with CH_2Cl_2 (3 x 150 mL). The combined organic phases were dried using a phase-separator and concentrated under reduced pressure. The crude product was purified by silica-gel flash column chromatography (10 – 33% EtOAc/heptane) to give aldehyde **S4**.

Yield: 1.22 g (89%). Obtained as a white crystalline solid, >95% by NMR spectroscopy and a single spot by TLC.

R_f: 0.19 (33% EtOAc/petroleum ether). Stains red with anisaldehyde stain. Emits red fluorescence under LW-UV (365 nm) after staining with anisaldehyde.

^{1}H NMR (400 MHz, $CDCl_3$): δ 9.92 (s, 1H), 6.80 (d, J = 7.9 Hz, 1H), 6.76 – 6.72 (m, 2H), 3.858 (s, 3H), 3.856 (s, 3H), 3.05 (app. s, 4H), 2.60 (s, 3H) ppm.

^{13}C{^{1}H} NMR (100 MHz, $CDCl_3$): δ 185.3, 163.1, 156.4, 149.1, 147.8, 134.9, 132.6, 120.3, 111.7, 111.4, 56.05, 55.96, 32.7, 30.2, 11.7 ppm.

FTIR (ATR): 3056 (w), 3017 (w), 2848 (w), 1688 (s), 1603 (s), 1588 (m), 1513 (s), 1450 (m), 1420 (m), 1313 (s) cm^{-1}.

MP: 68.6 – 70.4 °C

HRMS-ESI (*m/z*): $[M + H]^+$ Calcd for $C_{15}H_{18}NO_4$ 276.1236; Found 276.1233.

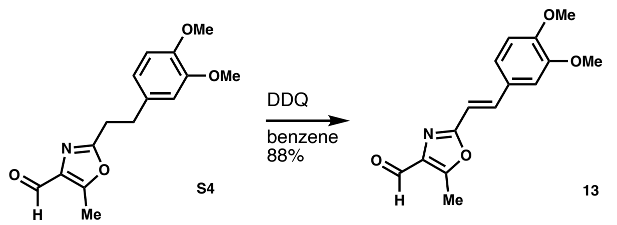

(*E*)-**2-(3,4-Dimethoxystyryl)-5-methyloxazole-4-carbaldehyde (13).**[4] A solution of aldehyde **S4** (520 mg, 1.90 mmol) in benzene (20 mL) was added to 2,3-dichloro-5,6-dicyano-1,4-benzoquinone (DDQ) (540 mg, 2.38 mmol) and the resulting dark green solution was heated to reflux with stirring for 20 h, at which point ^{1}H NMR spectroscopy indicated full consumption of the aldehyde starting material. The mixture was allowed to cool to room temperature, diluted with CH_2Cl_2 (20 mL) and then poured on a mixture of brine (20 mL) and sodium hydroxide (20 mL, 1 M, aq.). The phases were separated, and the water phase was extracted with additional CH_2Cl_2 (3 x 20 mL), the combined organic phases were then dried using a phase-separator and concentrated under reduced pressure. The crude product was purified by silica gel flash column chromatography (8 – 45% EtOAc/heptane) to give aldehyde **13** as a white crystalline solid (456 mg, 88%), >95% by NMR spectroscopy and a single spot by TLC. ^{1}H and ^{13}C{^{1}H} NMR spectroscopy data were in agreement with those previously reported.

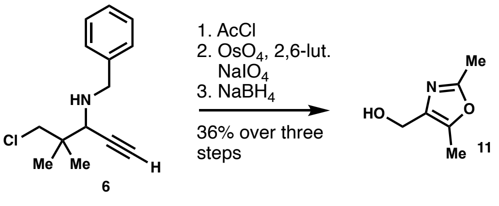

(2,5-Dimethyloxazol-4-yl)methanol (11).[5] To a solution of propargyl amine **6** (567 mg, 2.41 mmol) in CH_3CN (4.0 mL) was added acetyl chloride (165 µl, 2.32 mmol). The resulting solution was heated to 220 °C using microwave irradiation.[6] After 5 h, quantitative ^{1}H NMR spectroscopy using DMF as an internal standard indicated the formation of 1.20 mmol (50%) of the desired 4-alkenyl oxazole intermediate. The crude mixture was diluted with Et_2O (15 mL), washed with $NaHCO_3$ (3.0 x 15 mL, sat. aq.) and brine (15 mL). The organic phase was dried using a phase-separator and a short plug of silica and then carefully concentrated under

---

[4] Known compound: J. Linder; A. J. Blake; C. J. Moody *Org. Biomol. Chem.* **2008**, *6*, 3908.
[5] Known compound: M. J. Kukla; J. M. Fortunato *J. Org. Chem.* **1984**, *49*, 5003.
[6] The addition of PPh_3 leads to shortened reaction times (1 h at 220 °C) with a maintained yield. For purification reasons, it was however found superior to omit the PPh_3 additive.

reduced pressure.[7] The resulting oil was dissolved in dioxane:water (3:1, 10 mL) followed by the addition of 2,6-lutidine (280 µl, 2.40 mmol) and OsO_4 (480 µl, 2.5% in *t*-BuOH, 47 µmol). The resulting brown solution was stirred for 5 min at RT followed by the addition of sodium periodate (1024 mg, 4.79 mmol). The resulting suspension was stirred for 2 h at which point TLC indicated complete conversion of the alkenyl-oxazole intermediate. The reaction was quenched by the addition of $Na_2S_2O_3$ (3.0 mL, 300 g/l, aq.) and stirred for 1 h, then diluted with brine (40 mL) and extracted with CH_2Cl_2 (3 x 20 mL). The combined organic phases were dried using a phase-separator and the CH_2Cl_2 was carefully removed under reduced pressure. The crude mixture was diluted with a mixture of THF (4.0 mL) and isopropanol (4.0 mL) and cooled to 0 °C. To this solution was added $NaBH_4$ (31 mg, 0.81 mmol) and the resulting mixture was stirred at 0 °C for 20 min, at which point TLC indicated complete conversion of the oxazole-4-carbaldehyde intermediate. The reaction was quenched by the addition of brine (10 mL) and HCl (1 M) to pH 5 followed by stirring at RT for 1 h. The mixture was then diluted with water (5.0 mL), the phases were separated and the water phase was extracted with CH_2Cl_2 (10 x 10 mL). The combined organic phases were dried using a phase-separator and concentrated under reduced pressure. The crude product was purified by silica gel flash column chromatography in (50 – 100% Et_2O/heptane) to give alcohol **11** (105 mg, 36% over three steps) as an off-white solid, >95% by NMR spectroscopy and a single spot by TLC. ^{1}H and $^{13}C\{^{1}H\}$ NMR spectroscopy data were in agreement with those previously reported.

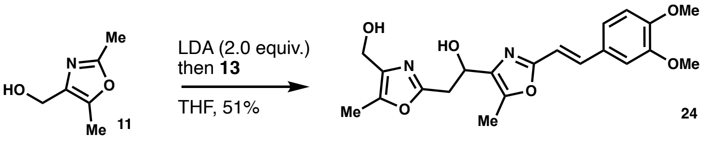

(*E*)-1-(2-(3,4-Dimethoxystyryl)-5-methyloxazol-4-yl)-2-(4-(hydroxymethyl)-5-methyloxazol-2-yl)ethanol (24). To a stirred solution of alcohol **11** (163 mg, 1.28 mmol) in

[7] The alkenyl-oxazole intermediate is volatile, which reflected in a reduced isolated yield.

THF (12 mL) at -78 °C was added a solution of LDA (2.56 mmol, freshly prepared) in THF (2.0 mL) dropwise over 5 min. Additional THF (0.50 mL) was used to complete the transfer. The resulting dark brown solution was stirred at -78 °C for 30 min at which point a pre-cooled (-78 °C) solution of aldehyde **13** (350 mg, 1.28 mmol) in THF (12 mL) was added drop-wise via cannula over 15 min followed by additional THF (2.0 mL) to complete the transfer. The resulting bright red solution was stirred at -78 °C for 5 h, and then quenched by addition of NH_4Cl (15 mL, aq. sat.) and water (5.0 mL). The resulting mixture was warmed to RT and diluted with brine (60 mL) and extracted with CH_2Cl_2 (3 x 60 mL). The combined organic phases were washed with brine (30 mL), dried using a phase separator then concentrated under reduced pressure. The crude product was purified by silica-gel flash column chromatography (0 – 5% CH_2Cl_2/MeOH) to give bis-oxazole **24**. Unreacted aldehyde **13** (46 mg, 13%), and alcohol **11** (19 mg, 12%) were also isolated. ^{1}H NMR spectroscopy of the crude product mixture indicated formation of a single regioisomeric product.

Yield: 239 mg (51%). Obtained as a yellow amorphous solid, >95% pure by NMR spectroscopy, and a single spot by TLC.

R_f: 0.14 (5% MeOH/CH_2Cl_2). Stains yellow with anisaldehyde stain.

^{1}H NMR (400 MHz, $CDCl_3$): δ 7.31 (d, *J* = 16.3 Hz, 1H), 7.06 – 6.99 (m, 2H), 6.83 (d, *J* = 8.2 Hz, 1H), 6.70 (dd, *J* = 16.3, 1.5 Hz, 1H), 5.10 (dd, *J* = 9.1, 4.0 Hz, 1H), 4.91 br. s, 1H), 4.46 (s, 2H), 3.90 (s, 3H), 3.88 (s, 3H), 3.32 (dd, *J* = 15.8, 9.2 Hz, 1H), 3.10 (dd, *J* = 15.8, 4.0 Hz, 1H), 2.34 (s, 3H), 2.26 (s, 3H) ppm.

^{13}C{^{1}H} NMR (100 MHz, $CDCl_3$): δ 160.8, 160.1, 150.2, 149.3, 145.1, 144.4, 136.7, 135.3, 133.9, 128.8, 121.2, 111.9, 111.3, 109.0, 64.5, 56.05, 55.96, 55.8, 35.4, 10.7, 10.2 ppm.

FTIR (ATR): 3243 (br. m), 2954 (w), 2924 (w), 1637 (m), 1512 (s), 1262 (s) cm^{-1}.

HRMS-ESI (*m*/*z*): $[M + H]^+$ Calcd for $C_{21}H_{25}$N2O_6 401.1713; Found 401.1705.

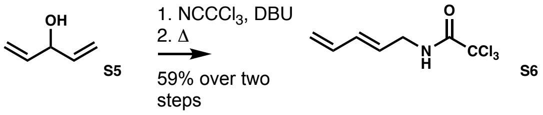

(*E*)-2,2,2-Trichloro-*N*-(penta-2,4-dien-1-yl)acetamide (S6).[8] To a stirred solution of 1,4-pentadien-3-ol **S5** (6.9 ml, 60 mmol) in CH_2Cl_2 (150 mL) was added 1,8-diazabicyclo(5.4.0)undec-7-ene (DBU) (1.8 mL, 13 mmol) dropwise over 3 min at 0 °C, followed by dropwise addition of trichloroacetonitrile (10.2 mL, 101 mmol) over 10 min. During the course of addition, the color gradually changed from pale yellow to dark brown. The mixture was stirred at 0 °C for 2 h, at which point no further change could be observed by TLC. The reaction mixture was then concentrated under reduced pressure and the resulting oily residue was diluted with 25% Et_2O/petroleum ether (1:4) and filtered through a short plug of silica (~2 x 2 cm) to remove an insoluble black tar. The filtrate was concentrated under reduced pressure to give the crude imidate as a yellow liquid that solidified in the freezer. The crude imidate was dissolved in toluene (200 mL) followed by the addition of K_2CO_3 (8.0 g, 58 mmol) and the resulting suspension was heated to reflux for 6 h at which point TLC indicated full conversion of the imidate intermediate (R_f: 0.26 (33% EtOAc/heptane), stains gray with anisaldehyde stain). The reaction mixture was allowed to cool to RT, the solids were removed by filtration and the filtrate was concentrated under reduced pressure. The resulting yellow oil was purified by silica gel flash column chromatography (250 g silica) eluting with 50% CH_2Cl_2/heptane (ca. 1 L), thereafter continuing with a gradually increasing amount of EtOAc (1 – 3%) added to the eluent to give amide **S6** (8.24 g, 59%) as a yellow oil, >95% by NMR spectroscopy and a single spot by TLC. ^1H and $^{13}C\{^1H\}$ NMR spectroscopy data were in agreement with those previously reported.

[8] Known compound, the protocol was adapted from: M. N. Paddon-Row; A. I. Longshaw; A. C. Willis; M. S. Sherburn *Chem. Asian J.* **2009**, *4*, 126.

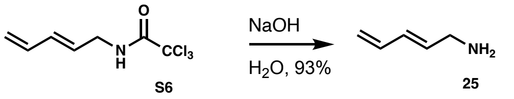

(*E*)-Penta-2,4-dien-1-amine (25).[8] Sodium hydroxide (20 mL, aq. 3M, 60 mmol) was added to (*E*)-2,2,2-trichloro-*N*-(penta-2,4-dien-1-yl)acetamide **S6** (6.61 g, 29 mmol) and the mixture was stirred vigorously at RT for 14 h at which point TLC indicated full consumption of the amide starting material. The reaction mixture was extracted with Et_2O until TLC indicated that no amine remained in the water phase. The combined organic phases were washed with brine, dried over anhydrous sodium sulfate and concentrated with careful reduced pressure control (>200 mbar, 25 °C) to give a mixture of amine **25** and diethyl ether (2.73 g, molar ratio 80 : 20, 27 mmol **25**, 93%) as a yellow liquid. ^{1}H and ^{13}C{^{1}H} NMR spectroscopy data were in agreement with those previously reported.

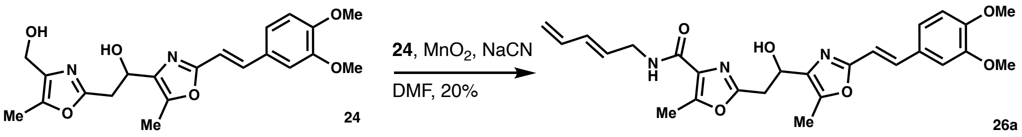

2-(2-(2-((*E*)-3,4-Dimethoxystyryl)-5-methyloxazol-4-yl)-2-hydroxyethyl)-5-methyl-*N*-((*E*)-penta-2,4-dien-1-yl)oxazole-4-carboxamide (26a). A mixture of diol **24** (30 mg, 75 μmol) and MnO_2 (88%, 518 mg, 5.2 mmol) was suspended in DMF (2.0 mL). To the suspension was added NaCN (37 mg, 0.75 mmol) followed by amine **25** (25 mg, 80%$_{mass}$ in Et_2O, 0.24 mmol). The suspension was stirred for a 20 h, then filtered through a short plug of silica (~2 x 2 cm) eluting with 10% MeOH/EtOAc, and concentrated. The crude product was purified by silica gel flash column chromatography in (25 – 33% EtOAc/heptane) to give amide **26a**.

Yield: 7.4 mg (20%). Obtained as a pale yellow amorphous solid, >95% pure by NMR spectroscopy, and a single spot by TLC.

R*f*: 0.20 (33% EtOAc/heptane). Stains blue with anisaldehyde stain.

^{1}H NMR (400 MHz, C_6D_6): δ 7.54 (d, *J* = 16.3 Hz, 1H), 7.00 (br. s, 1H), 6.94 – 6.80 (m, 3H), 6.44 (d, *J* = 8.3 Hz, 1H), 6.25 – 6.10 (m, 1H), 6.09 – 5.98 (m, 1H), 5.54 – 5.39 (m, 1H), 5.26 (s, 1H), 5.01 (d, *J* = 16.9 Hz, 1H), 4.91 (d, *J* = 10.1 Hz, 1H), 4.15 (br. s, 1H), 3.95 – 3.81 (m, 2H), 3.51 – 3.22 (m, 8H), 2.46 (s, 3H), 2.11 (s, 3H) ppm.

^{13}C{^{1}H} NMR (101 MHz, $CDCl_3$): δ 161.7, 160.2, 159.3, 153.3, 150.3, 149.3, 144.4, 136.2, 136.2, 135.5, 133.1, 129.6, 129.0, 128.7, 121.2, 117.7, 111.9, 111.3, 109.0, 64.5, 56.1, 56.0, 40.5, 35.4, 11.7, 10.7 ppm.

HRMS-ESI (*m/z*): $[M + H]^+$ Calcd for $C_{26}H_{30}N_3O_6$ 480.2135; Found 480.2137.

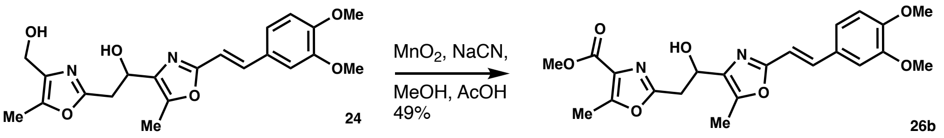

(*E*)-Methyl-2-(2-(2-(3,4-dimethoxystyryl)-5-methyloxazol-4-yl)-2-hydroxyethyl)-5-methyloxazole-4-carboxylate (26b).[9] A suspension of diol **24** (21.5 mg, 0.054 mmol), manganese dioxide (150 mg, 88%, 1.5 mmol), sodium cyanide (10.5 mg, 0.21 mmol), and acetic acid (10 µl, 0.17 mmol) in MeOH (0.30 mL) was stirred at RT for 2 days, at which point an additional portion of MnO_2 (100 mg, 1.0 mmol) was added. The resulting suspension was stirred for an additional 5 days at which point TLC indicated that the reaction did not proceed further. The mixture was then filtered through a plug of celite eluting with 10% MeOH/EtOAc, and the filtrate was concentrated under reduced pressure. The crude product was purified by silica gel flash-column chromatography (0 – 50% EtOAc/heptane) to give ester **26b** (11.2 mg, 49%) as a white solid, >95% pure by NMR spectroscopy and a single spot by TLC. ^{1}H and ^{13}C{^{1}H} NMR spectroscopy data were in agreement with those previously reported.

[9] Known compound, see reference 4.

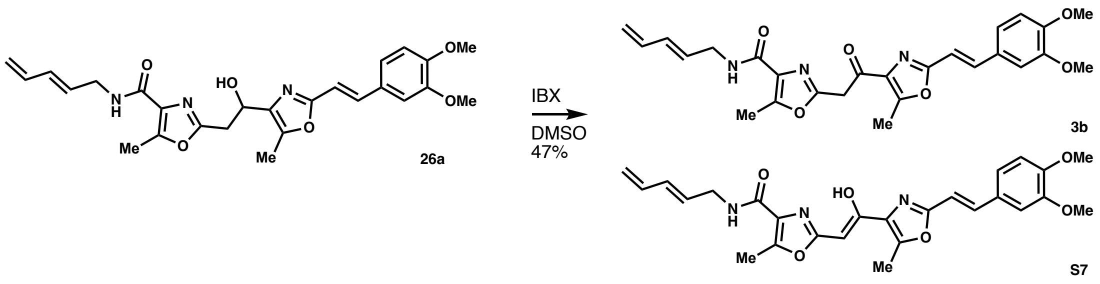

Siphonazole B (3b).[10] A solution of hydroxy-amide **26a** (17.0 mg, 0.0355 mmol) and IBX (29.8 mg, 0.106 mmol) in DMSO (2.0 mL) was stirred at RT for 4.5 h. Upon complete consumption of **26a** (TLC control), the solution was diluted with EtOAc (10 mL), washed with $NaHCO_3$ (3.0 x 10 mL, sat. aq.), and brine (10 mL). The organic phase was dried using a phase-separator and concentrated under reduced pressure. The crude product was purified by silica-gel flash column chromatography in (23 – 25% EtOAc/heptane) to give siphonazole B (**3b**). ^{1}H and ^{13}C{^{1}H} NMR spectroscopy data were in agreement with those previously reported.

Yield: 8.0 mg (47%). Obtained as a pale-yellow solid which was a single spot by TLC. NMR spectroscopy data shows a ~ 60:40 mixture of keto-enol tautomers (**3b** and **S7**) along with <5% impurities.

R*f*: 0.59 (25% EtOAc/heptane). Stains blue with anisaldehyde stain.

^{1}H NMR (500 MHz, acetone-d$_6$): δ 7.56 (br. t, *J* = 5.9 Hz, 1H), 7.51 (d, *J* = 16.4 Hz, 1H), 7.40 (d, *J* = 2.0 Hz, 1H), 6.43 – 6.34 (m, 1H), 6.28 – 6.22 (m, 1H), 5.82 (dt, *J* = 15.2, 6.0 Hz, 1H), 5.20 – 5.16 (m, 1H), 5.06 – 5.03 (m, 1H), 4.44 (s, 2H), 3.93 (s, 3H), 3.88 (s, 3H), 2.66 (s, 3H), 2.61 (s, 3H) ppm.

^{13}C{^{1}H} NMR (126 MHz, acetone-d$_6$): δ 190.2, 162.1, 160.2, 156.9, 156.0, 153.9, 152.1, 150.9, 138.3, 137.7, 135.4, 132.9, 132.0, 130.7, 129.2, 122.8, 117.1, 112.7, 111.7, 110.7, 56.3, 56.2, 40.8, 40.1, 12.4, 11.6 ppm.

[10] For spectroscopic data and assignment, see reference 4.

FTIR (ATR): 3356 (br), 2918 (w), 2849 (w), 1687 (m), 1650 (m), 1632 (m), 1509 (s) 1464 (m), 1420 (m), 1354 (m), 1262 (s), 1137 (s), 1022 (s) cm^{-1}.

HRMS-ESI (*m/z*): $[M + Na]^+$ Calcd for $C_{26}H_{27}N_3O_6Na$ 500.1798; Found 500.1795.

IV. Optimization of the oxazole-rearrangement

Table S1. Optimization of the rearrangement of oxazole **14** to 4-alkenyl oxazole **15.**

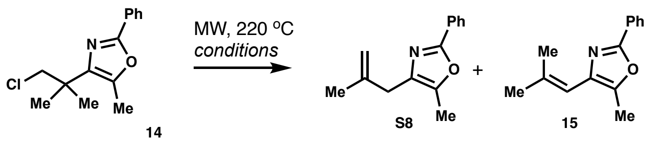

Solvent (conc. M)	*Additive (equiv.)*	*Time (/h)*	*Yield (%)*	
			**S8**	**15**
NMP (0.8)	PPh_3 (0.2)	3	N.d.	74
NMP (0.77)	PPh_3 (0.1)	4	N.d.	66
NMP (0.8)	-	3	32	64
NMP (1.3)	PPh_3 (0.2)	3	N.d.	58
CH_3CN (0.14)	PPh_3 (0.2)	7	N.d.	58[a]
CH_3CN (0.5)	PPh_3 (0.2)	4	N.d.	57[a]
CH_3CN (0.8)	-	3	23	56
NMP (0.4)	PPh_3 (0.2)	3	N.d.	51[a]
CH_3CN (0.08)	-	3	N.d.	n.d.
NMP (0.14)	-	1	N.d.	n.d.

General procedure: Reactions were performed by heating 0.5 – 1.0 mL of a solution of **14** and the additive indicated in a sealed vessel using microwave radiation to 220 °C. Yields were measured by ^{1}H NMR spectroscopy (20s relaxation delay) using mesitylene as an internal standard. a) Isolated yield. NMP = *N*-methyl pyrrolidine; MW = microwave irradiation. N.d. = not detected.

V. Optimization of the domino oxazole formation-rearrangement

Table S2. Optimization of the domino-reaction converting propargyl amine **6** to 4-alkenyl oxazole **12.**

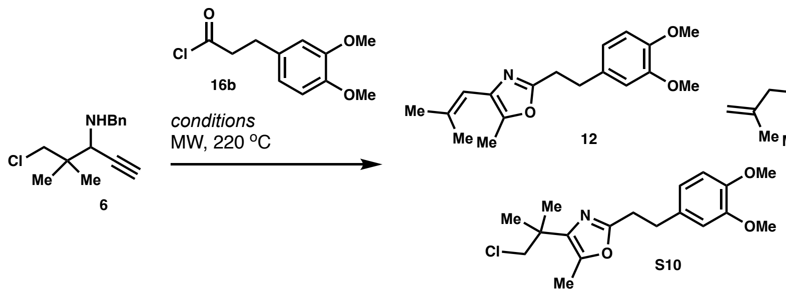

Solvent (conc./M)	*Additive (equiv.)*	*Temp. (℃)*	*Time (h)*	Yield **12 / S9 / S10** (%)
CH_3CN	PPh_3 (0.4)	220	1 h	59 / 4 / –
CH_3CN	PPh_3 (0.4)	220	40 min	55 / 3 / –
CH_3CN	PPh_3 (0.4)	220	20 min	55 / 5 / –
CH_3CN	PPh_3 (0.2)	220	45 min	50 / 5 / –
CH_3CN	PPh_3 (1.0)	220	20 min	50 / – / –
CH_3CN	DABCO (0.5)	220	50 min	41 / 5 / 9
CH_3CN	$(MeO)_3PO$ (0.2)	215	1 h	43 / – / 10
CH_3CN	DMAP (1.1)	220	1 h	19[a] / – / 10
dioxane	PPh_3 (0.4)	220	1 h	– / – / Only product
CH_3CN	-	220	1.5 h	49 / – / –
CH_3CN / $(MeO)_3PO$ (4:1)	-	220	1 h	Decomp.
$MeNO_2$	-	220	4 h	29 / 12 / 10
$MeNO_2$	-	220	1.5 h	15 / – / 20
EtOAc	-	220	1.5	– / – / 68
BMIM PF_6	-	150	4	15 / – / 24
DMF	-	220	1 h	23 / 35 / 0
NMP	-	220	1 h	11 / – / 35
DMSO	-	220	8 min	Decomp.
DMSO	-	180	1 h	No reaction
toluene	-	220	1.5 h	– / – / Only product

General procedure: The reactions were performed with 50 mg of **6** and 1.1. equiv. of acid chloride **16b** in 0.5 mL of the indicated solvent. The reactions were heated in a closed vessel by microwave irradiation. Yields were measured by ^1H NMR spectroscopy (20 s relaxation delay) using DMF or mesitylene as an internal standard. The yield for **12** includes *O*-demethylated 4-alkenyl oxazole products. a) Isolated yield. NMP = *N*-methyl pyrrolidine; BMIM = 1-butyl-3-methylimidazolium; DABCO = 1,4-diazabicyclo[2.2.2]octane; DMAP = 4-dimethylaminopyridine; MW = microwave irradiation.

*VI. Crystal structure data for aldehyde **S4***

Crystallization procedure: Single crystals of aldehyde **S4**, suitable for scXRD, were grown from a mixture of toluene and hexane with slow vapor diffusion.

Table S3. Crystal data and refinement results for aldehyde **S4**.

Chemical formula	$C_{15}H_{17}NO_4$	
Formula weight	275.29	
Collection temperature /K	293(2) K	
Crystal size /mm^3	0.2 x 0.2 x 0.05	
Crystal habit	colorless, plate	
Wavelength /Å	0.71073	
Crystal system	Monoclinic	
Space group	$P2_1/n$	
Unit cell dimensions:	a = 8.7488(9) Å	α = 90°
	b = 5.8898(6) Å	β = 91.903(10)°
	c = 26.974(3) Å	γ = 90°
Unit cell volume /Å^3	1389.2(3)	
Z, Calculated density /Mg/m^3	4, 1.316	
Radiation type	MoKα	
Absorption coefficient, m/mm^{-1}	0.096	
No. reflections collected / unique	11504 / 3275	
R_{int}	0.0393	
Completeness to theta = 25.000 /%	99.9	
Data / restraints / parameters	3275/ 0 / 184	
Goodness of fit on F^2	1.040	
Final R indices $(I > 2\sigma(I))$	R_1 = 0.0492, wR_2 = 0.1058	
R indices *(all data)*	R_1 = 0.0785, wR_2 = 0.1226	
Absolute structure parameter	–	
Largest diff. peak and hole /e-/Å^3	0.156 and -0.179	
CCDC	2286184	

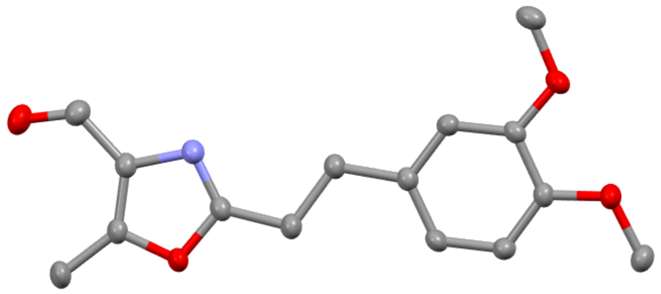

Figure S1. Asymmetric unit of aldehyde **S4**. Black = carbon atom; red = oxygen atom; blue = nitrogen atom. Thermal ellipsoids are shown at 30% probability. Hydrogen atoms are omitted for clarity.

*VI. NMR spectra for compounds **S2**, **9**, **6**, **14/15**, **16b**, **12**, **S4**, **13**, **11**, **24**, **S6**, **25**, **26a/b**, and **3b***

^{1}H NMR, $CDCl_3$, 400 MHz

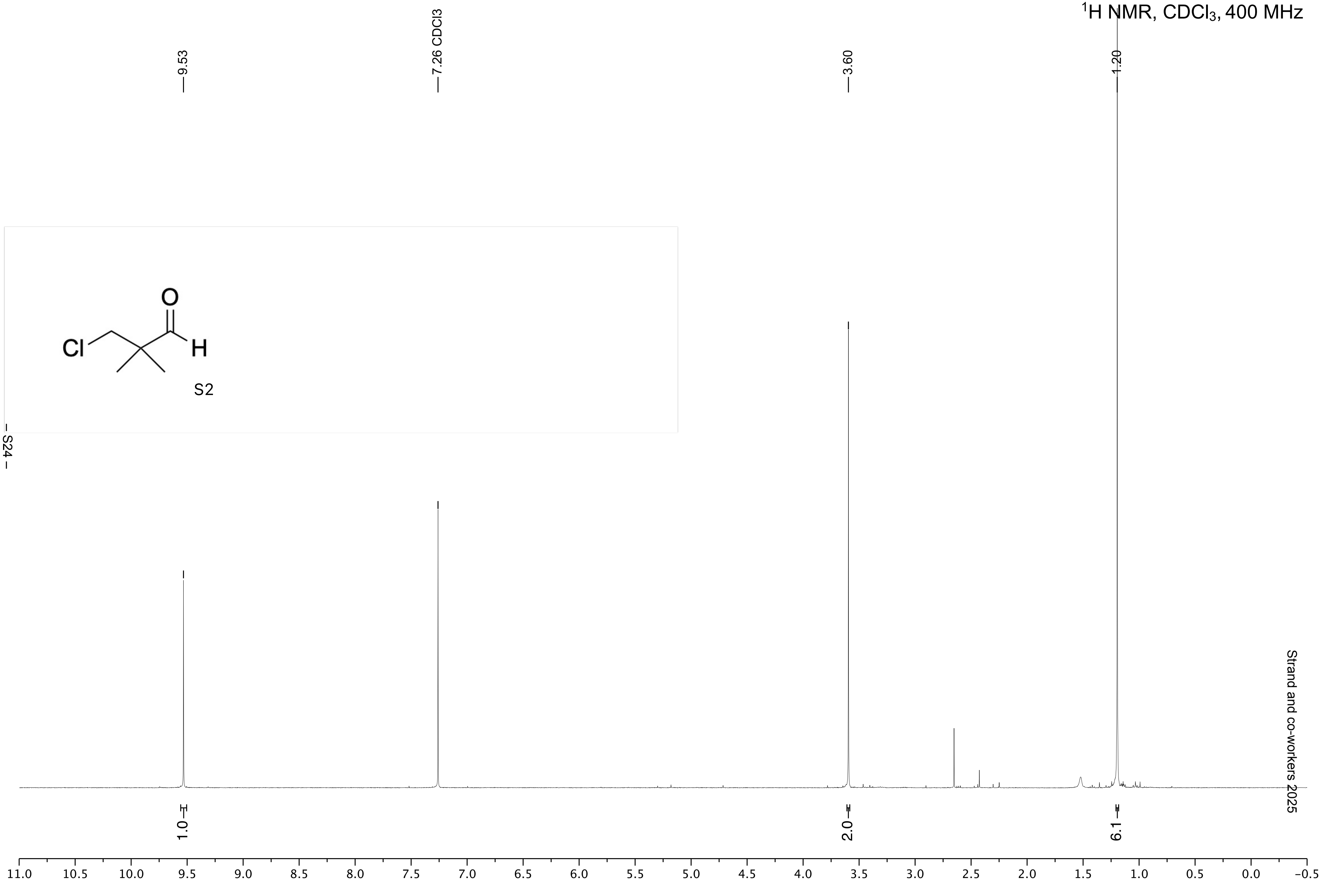

^1H NMR, $CDCl_3$, 400 MHz

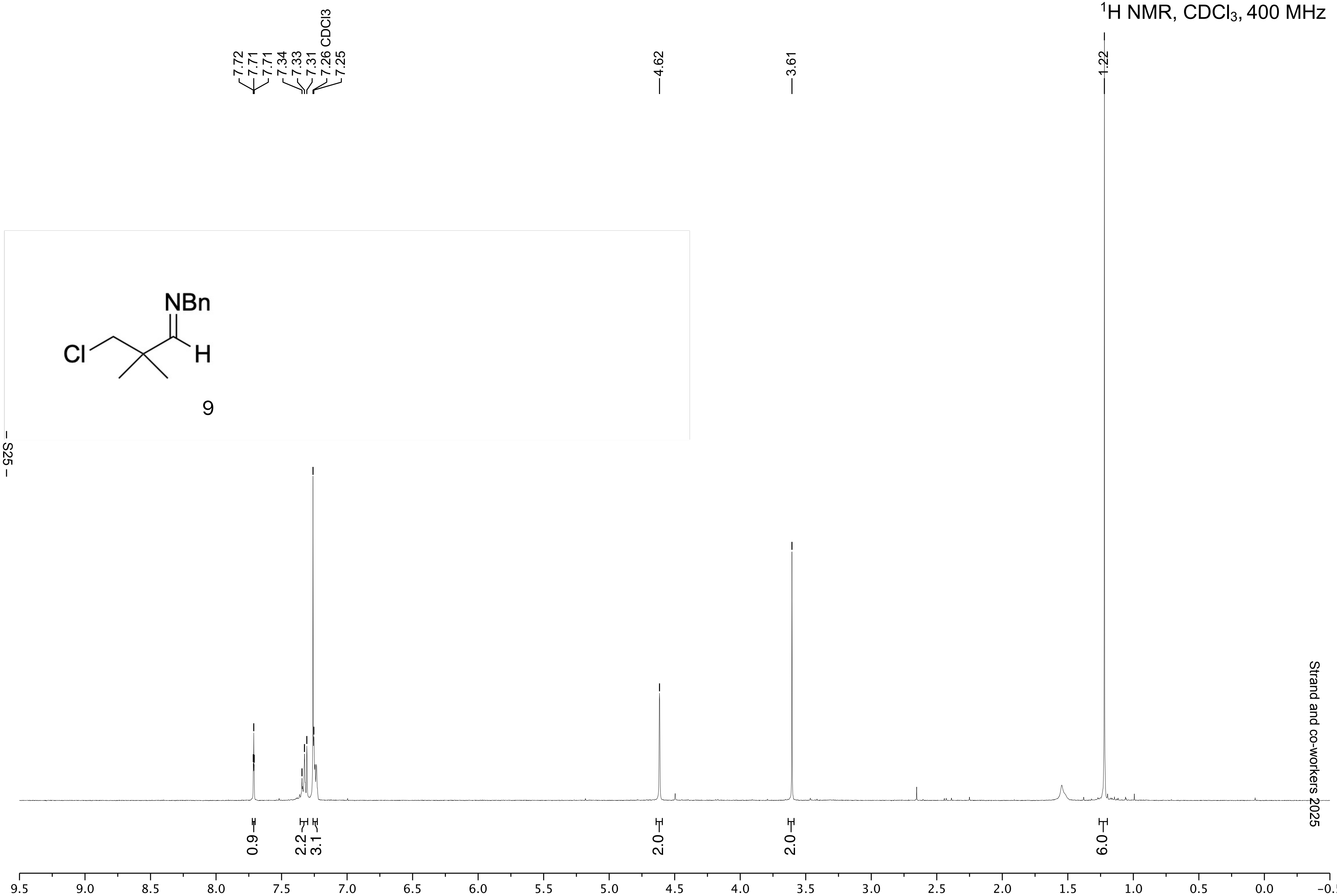

9

^{1}H NMR, $CDCl_3$, 400 MHz

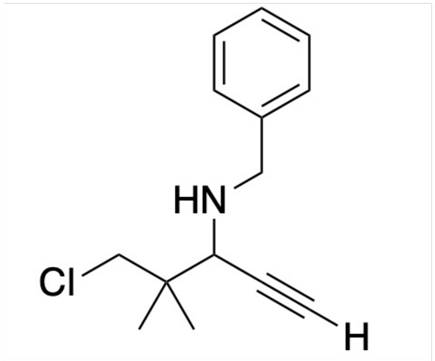

6

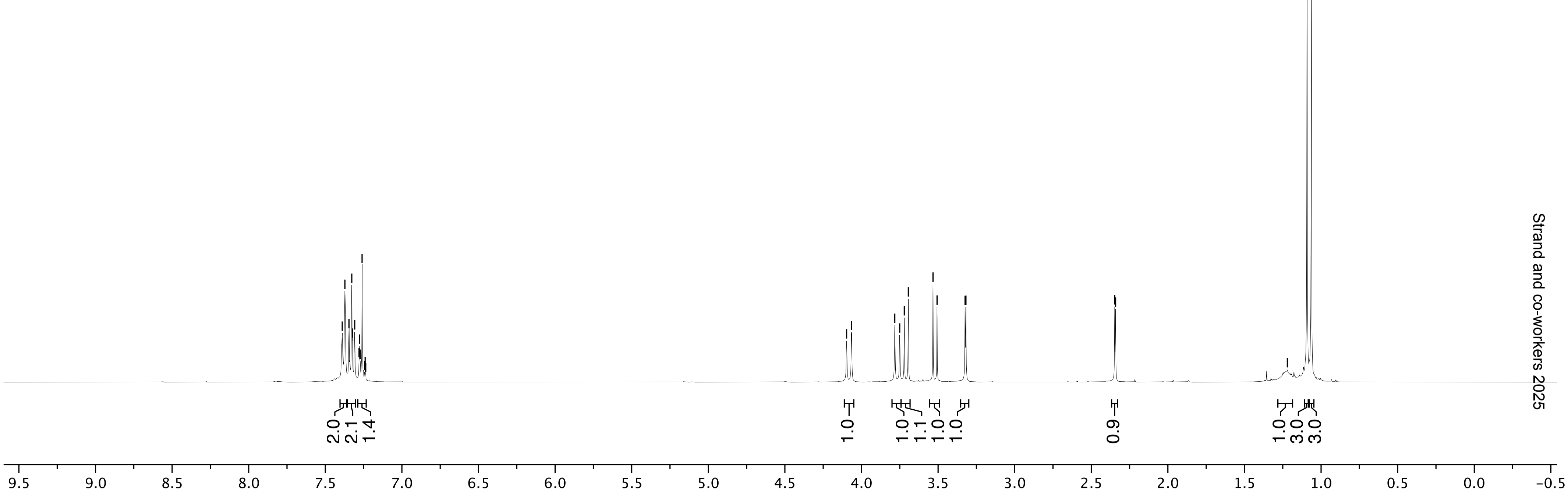

Strand and co-workers 2025

$^{13}C\{^1H\}$ NMR, $CDCl_3$, 101 MHz

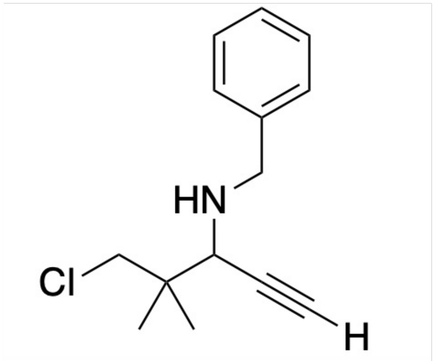

6

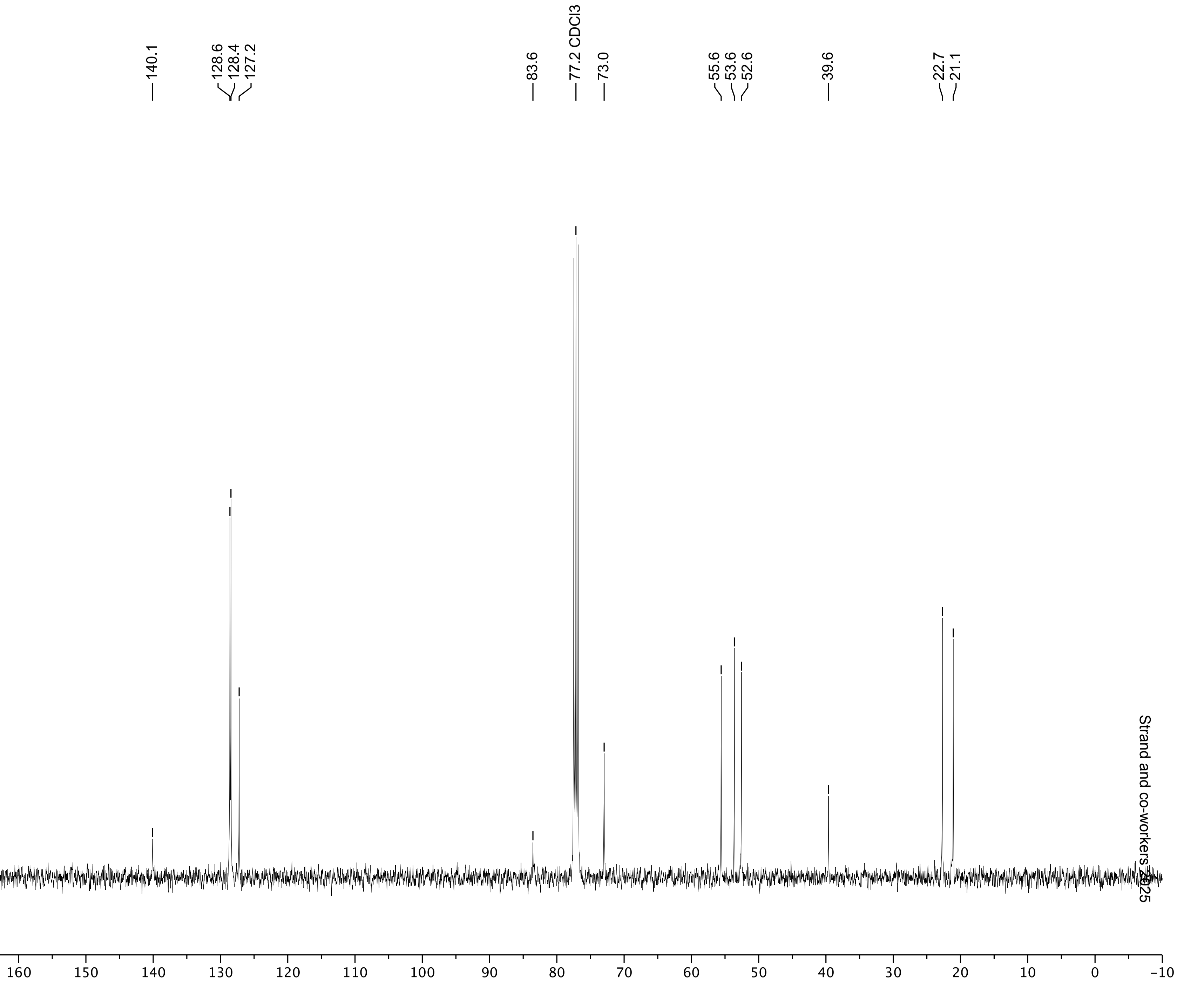

^{1}H NMR, $CDCl_3$, 400 MHz

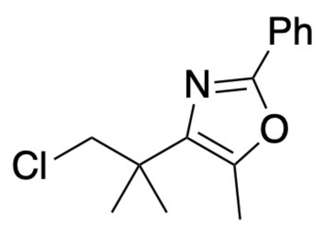

14

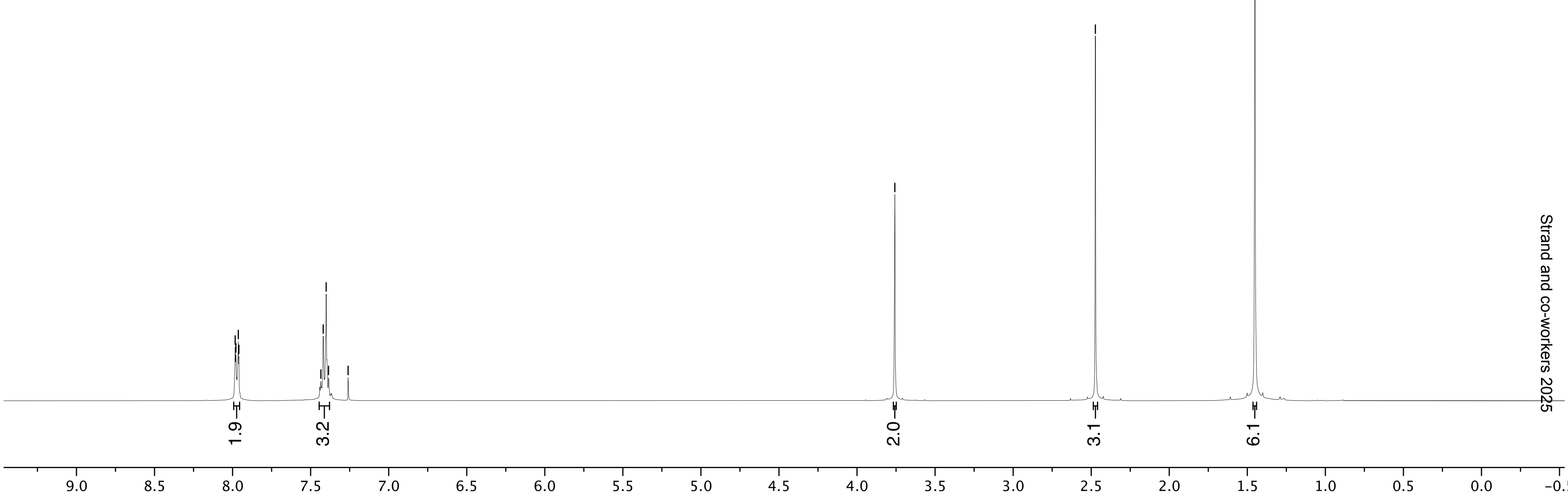

^1H NMR, $CDCl_3$, 400 MHz

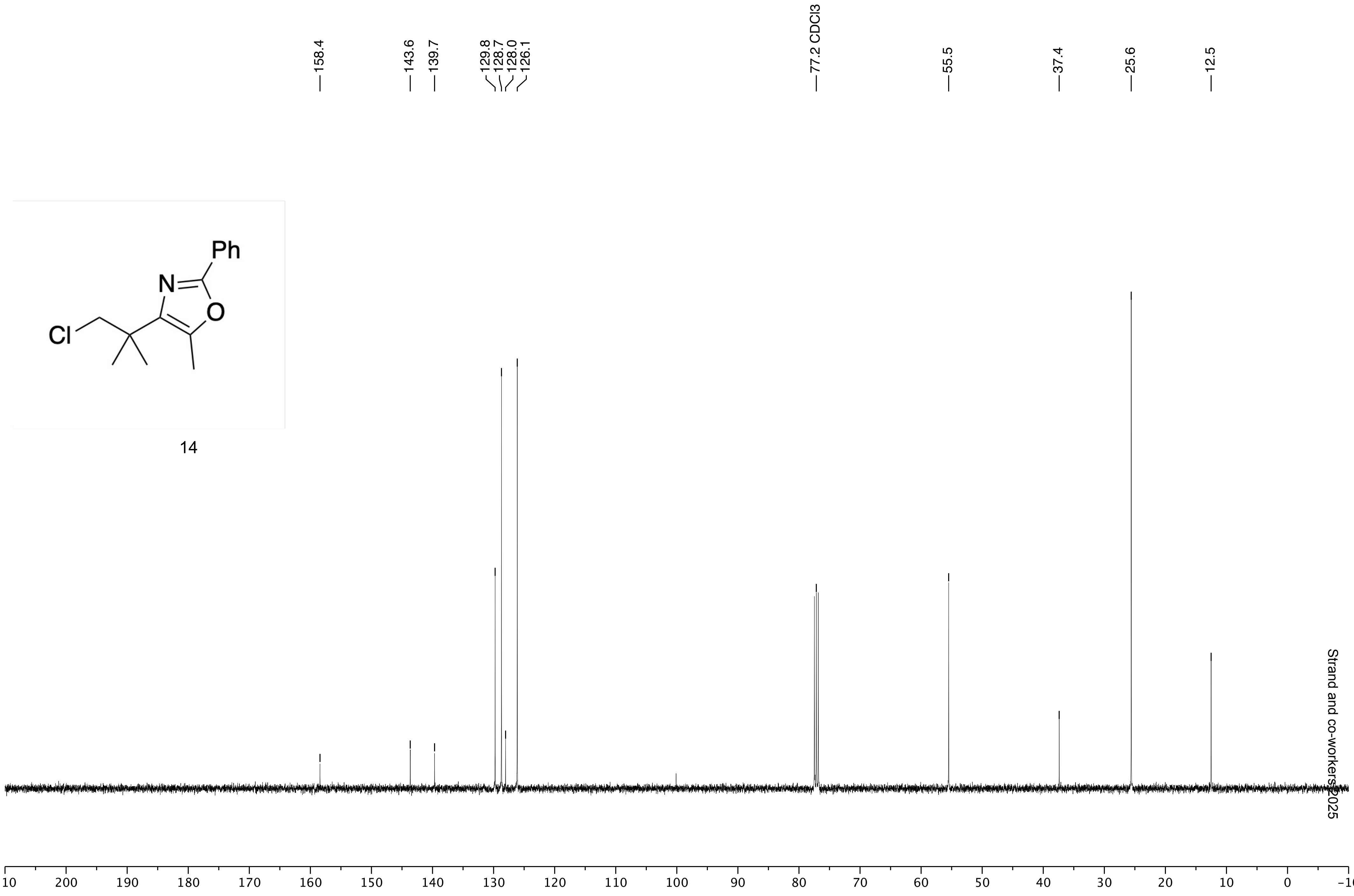

14

^{1}H NMR, $CDCl_3$, 400 MHz

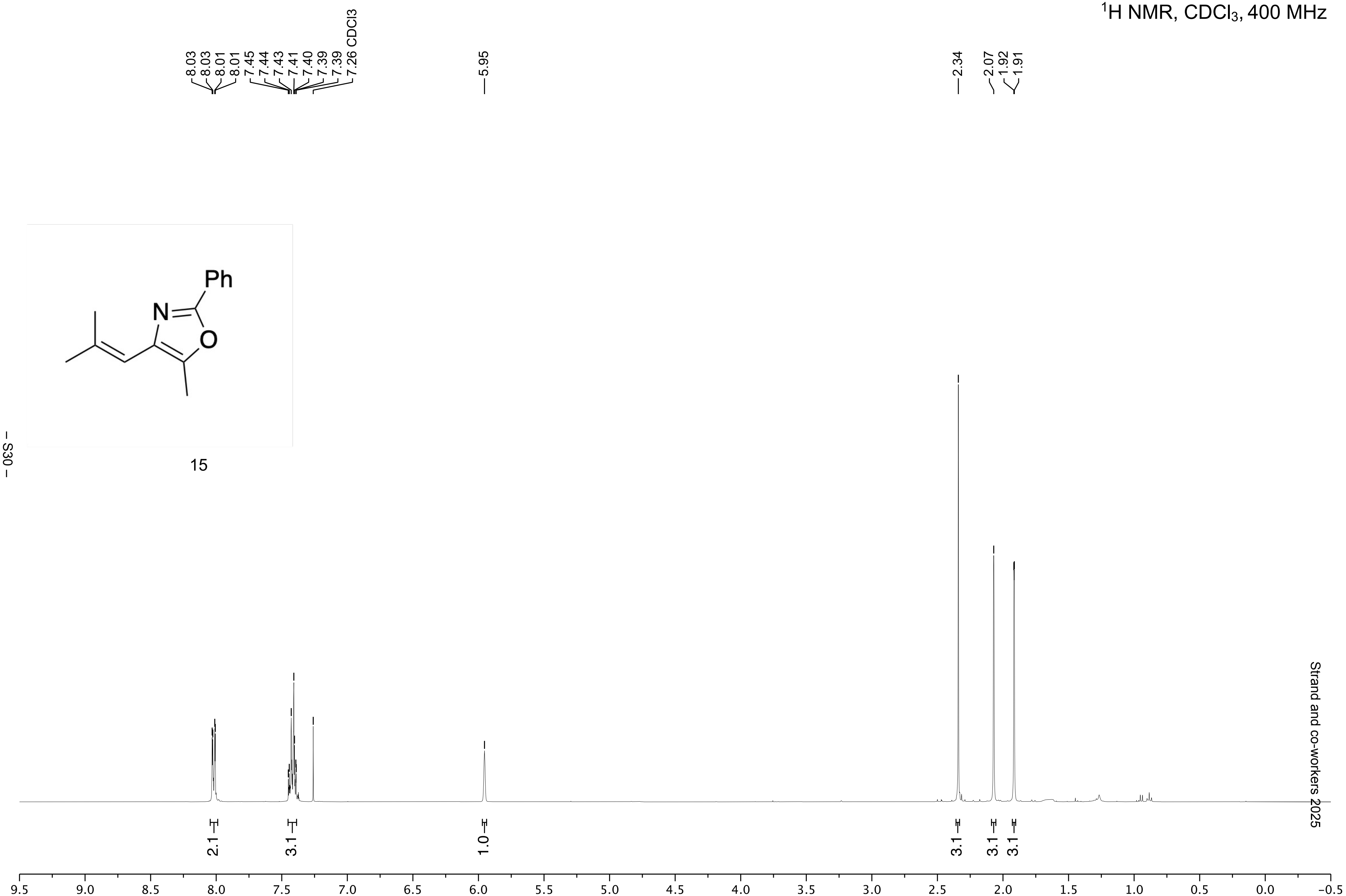

$^{13}C\{^{1}H\}$ NMR, $CDCl_3$, 101 MHz

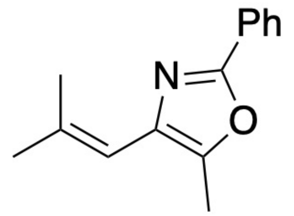

15

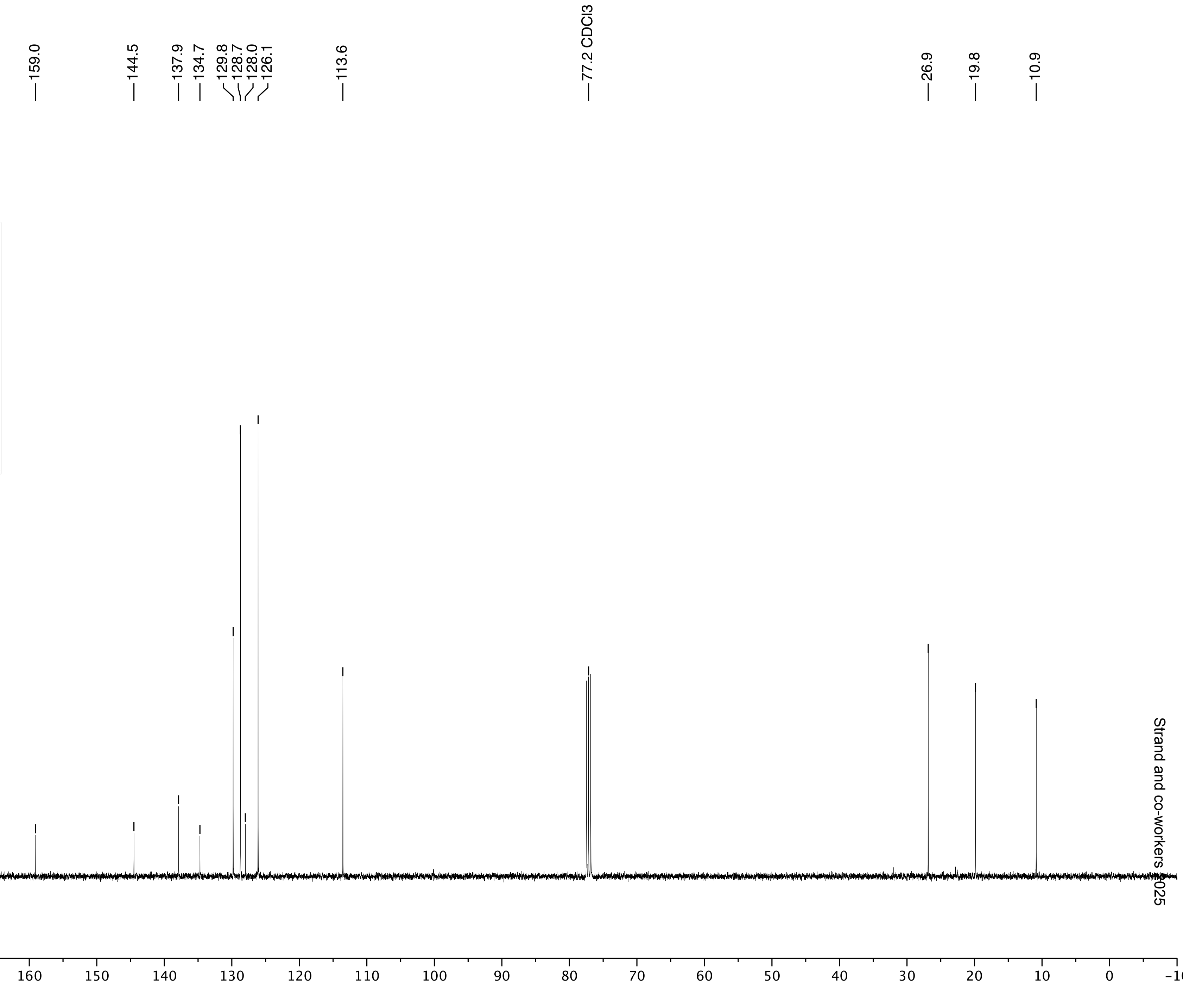

^{1}H NMR, $CDCl_3$, 400 MHz

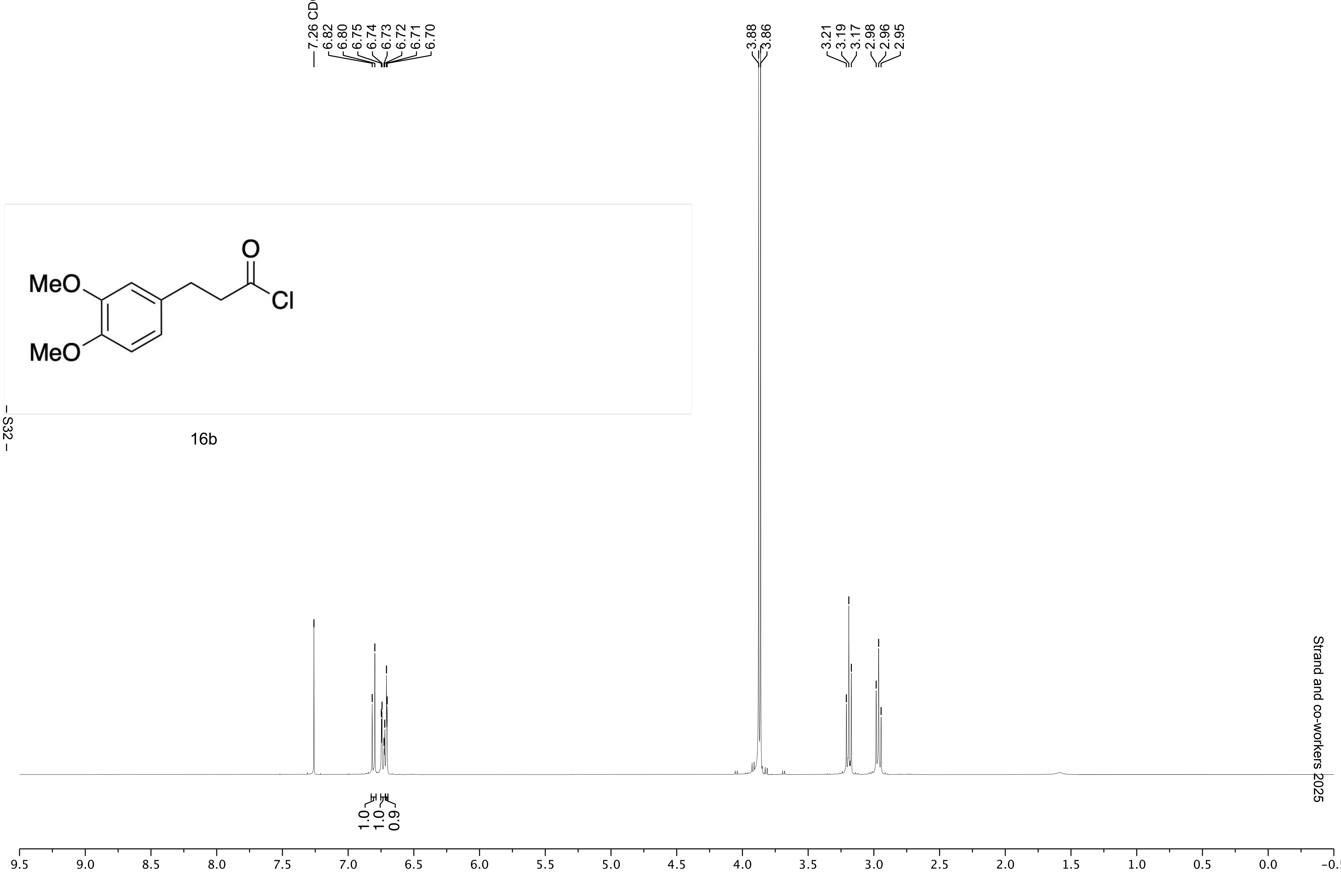

16b

$^{13}C\{^1H\}$ NMR, $CDCl_3$, 101 MHz

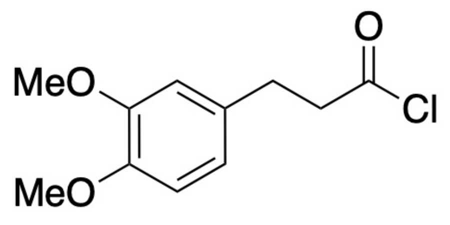

16b

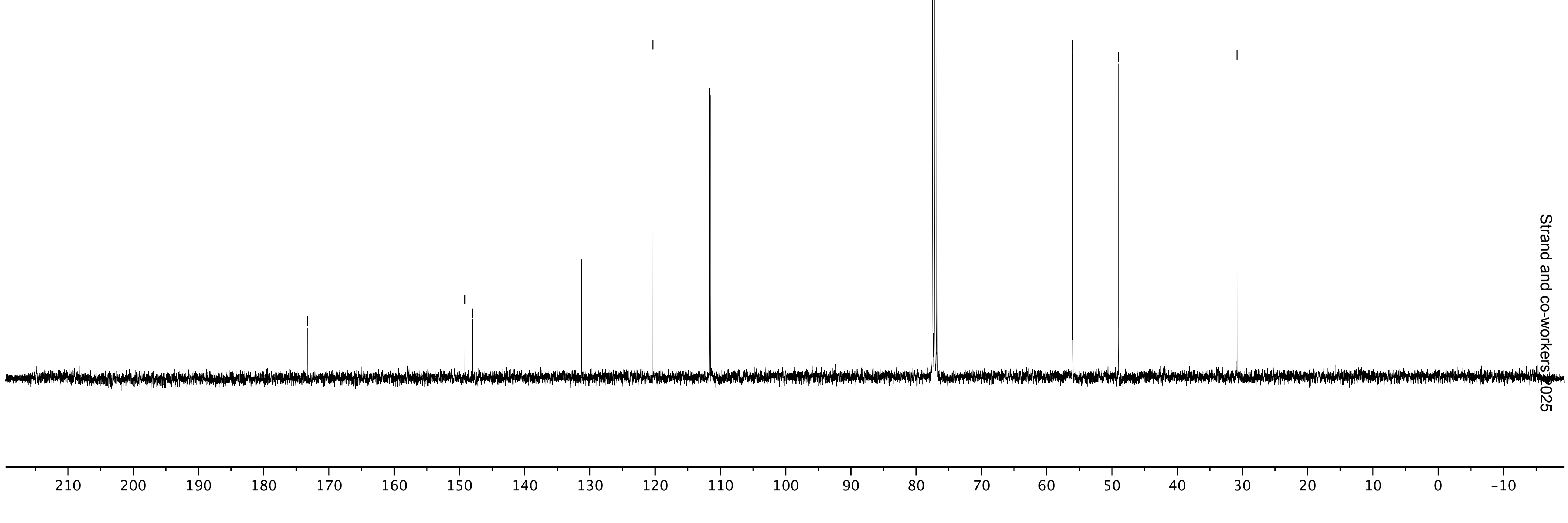

^1H NMR, $CDCl_3$, 400 MHz

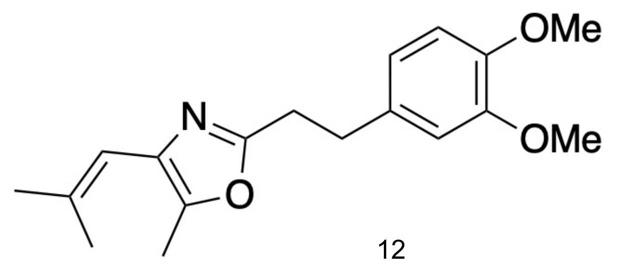

12

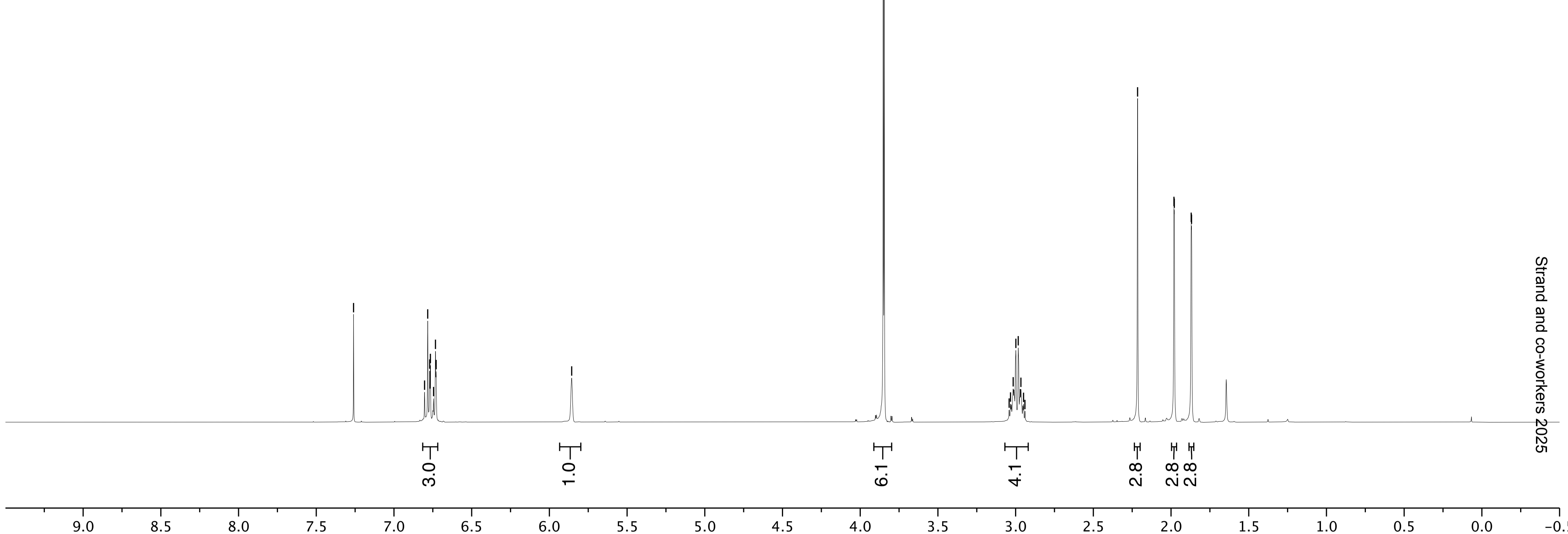

$^{13}C\{^1H\}$ NMR, $CDCl_3$, 101 MHz

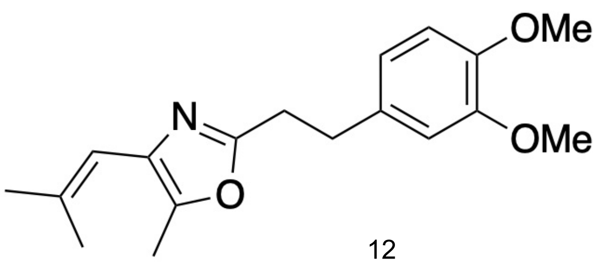

12

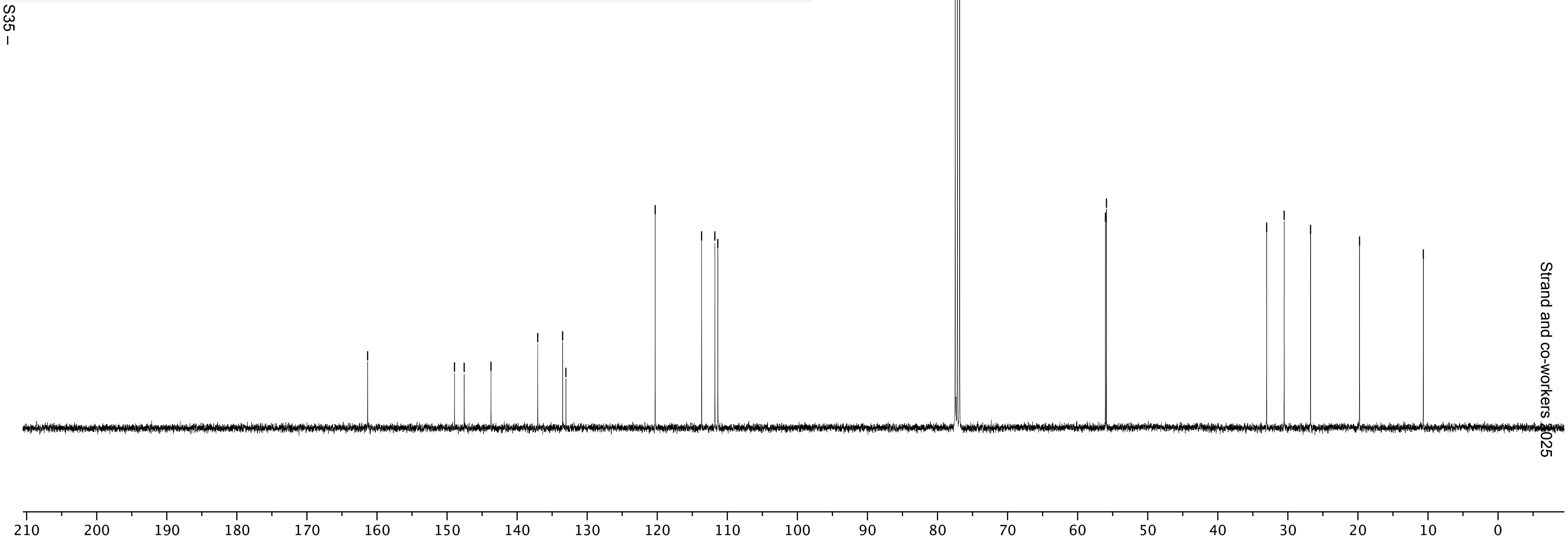

^1H NMR, $CDCl_3$, 400 MHz

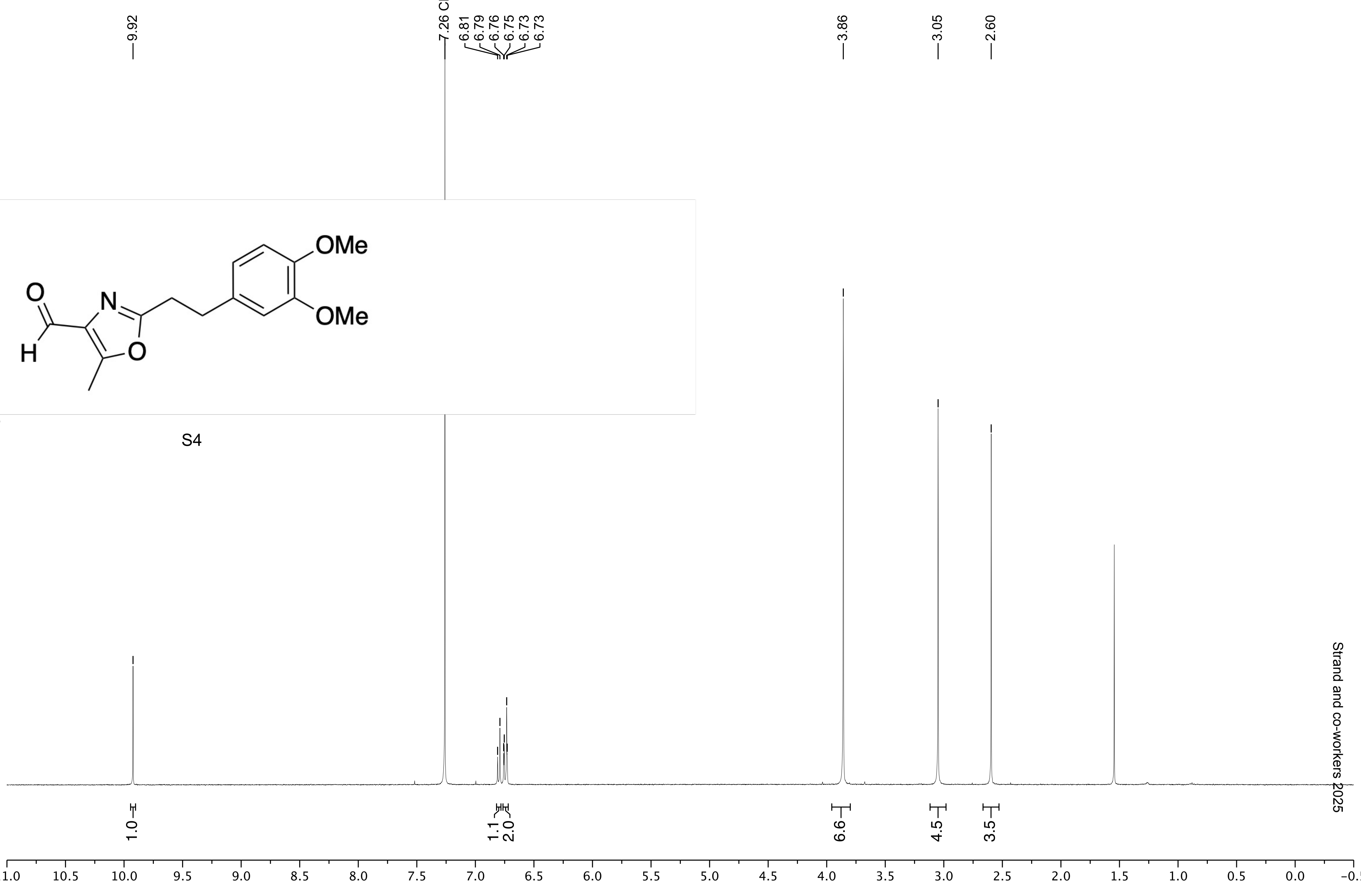

S4

$^{13}C\{^1H\}$ NMR, $CDCl_3$, 101 MHz

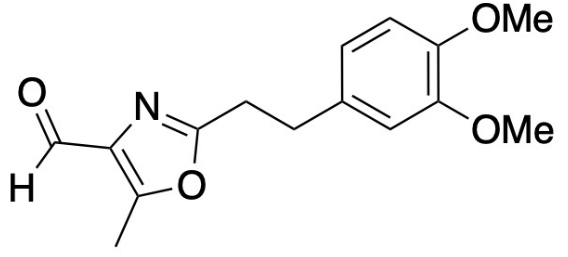

S4

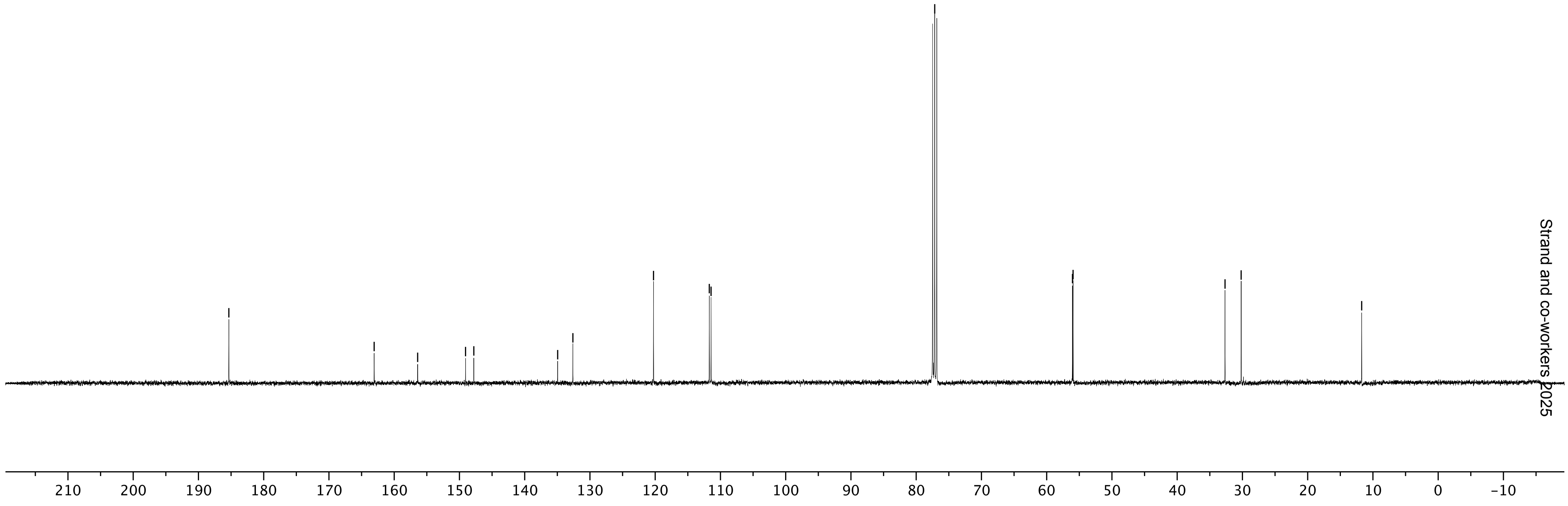

^{1}H NMR, $CDCl_3$, 400 MHz

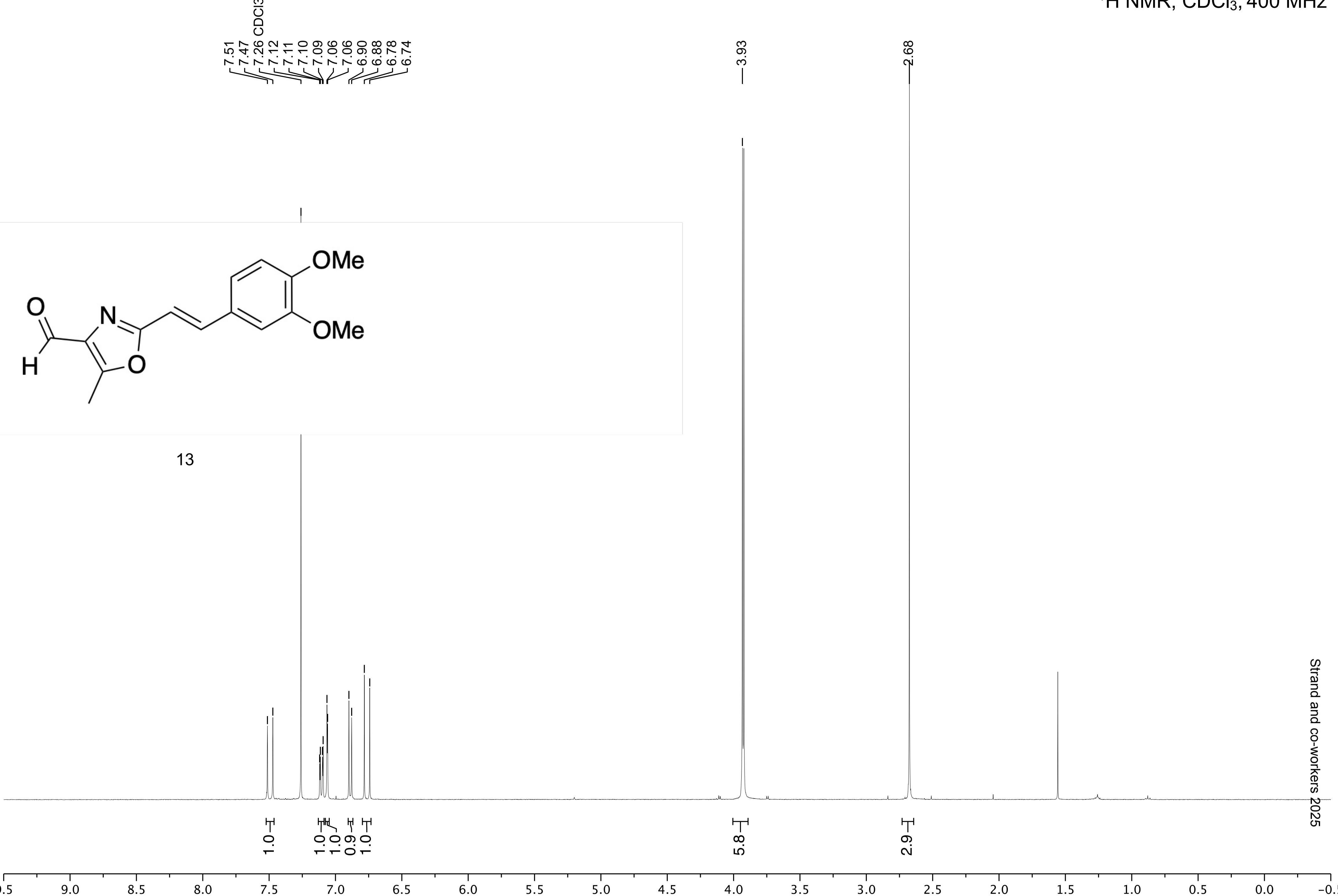

13

$^{13}C\{^1H\}$ NMR, $CDCl_3$, 101 MHz

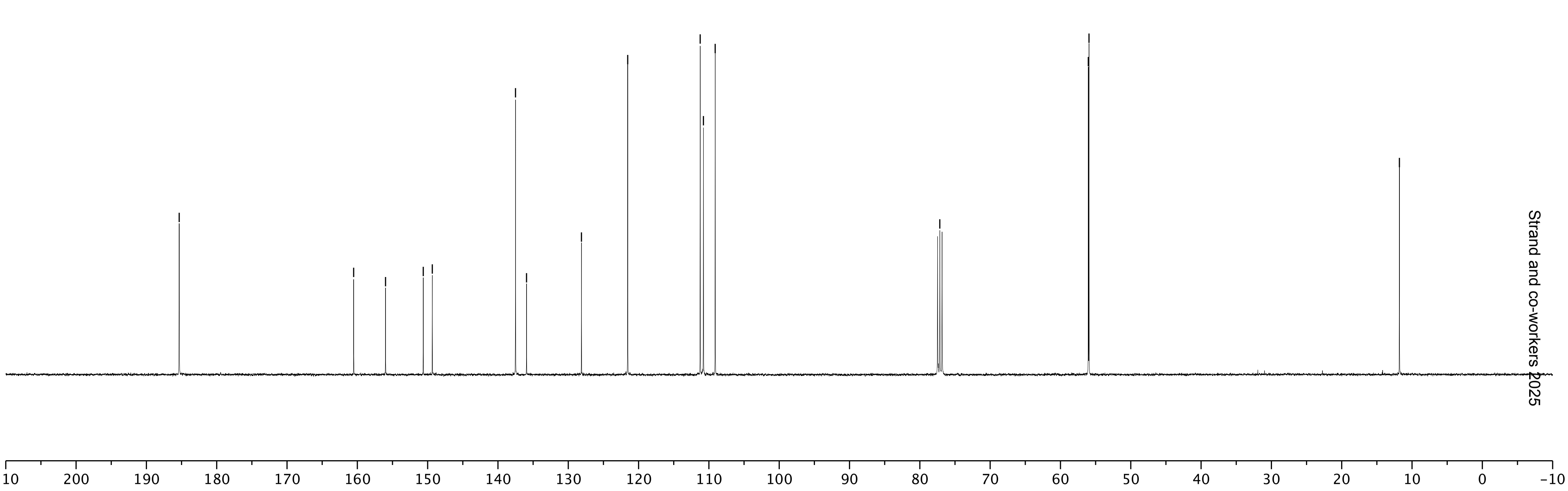

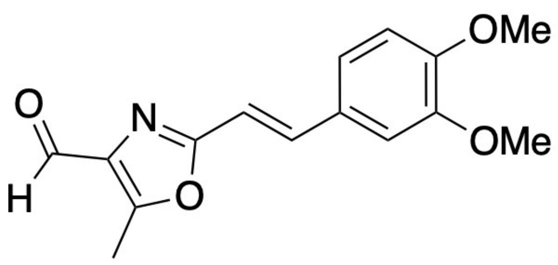

13

Strand and co-workers 2025

^1H NMR, $CDCl_3$, 400 MHz

11

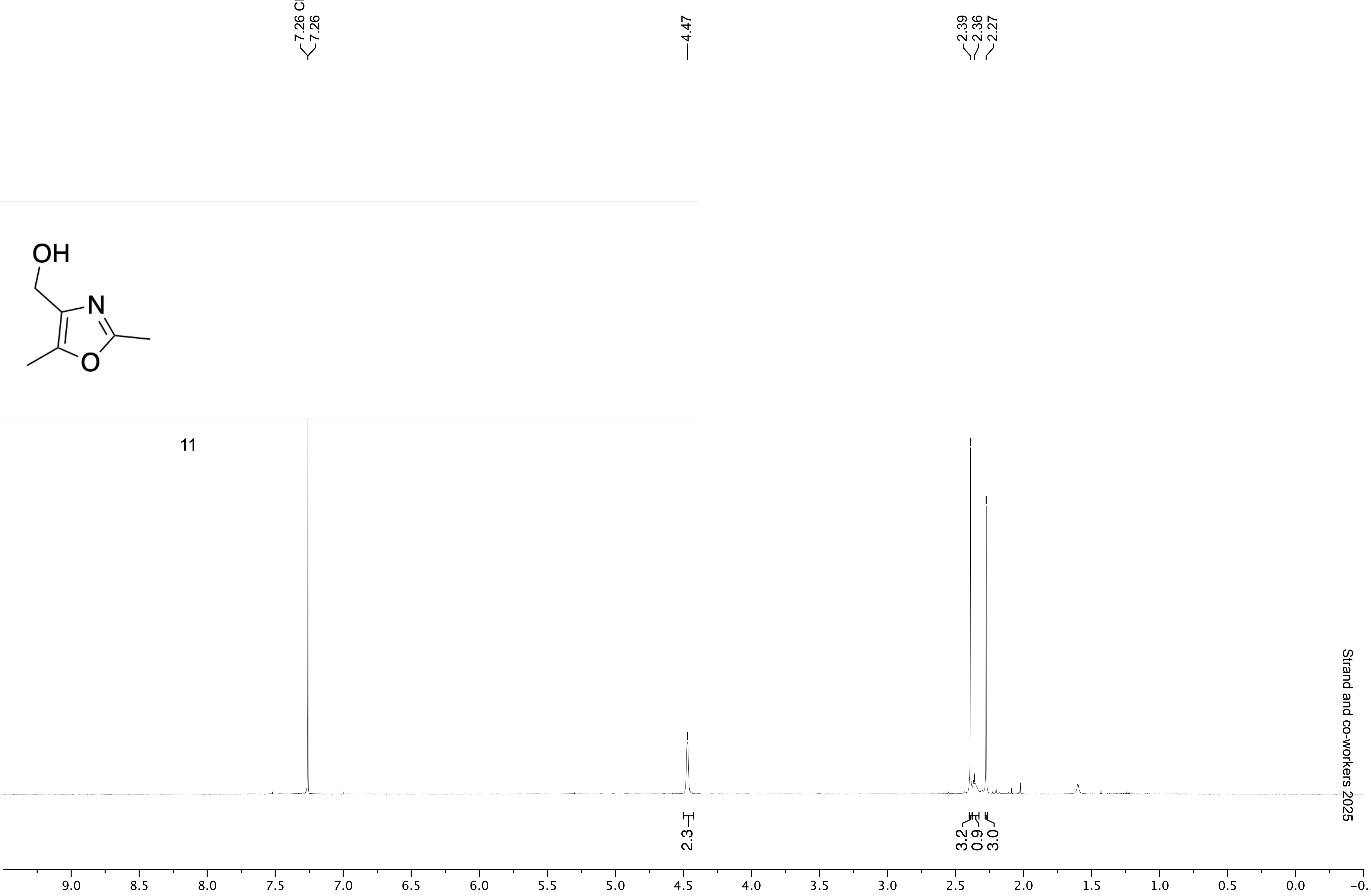

Strand and co-workers 2025

^{1}H NMR, $CDCl_3$, 400 MHz

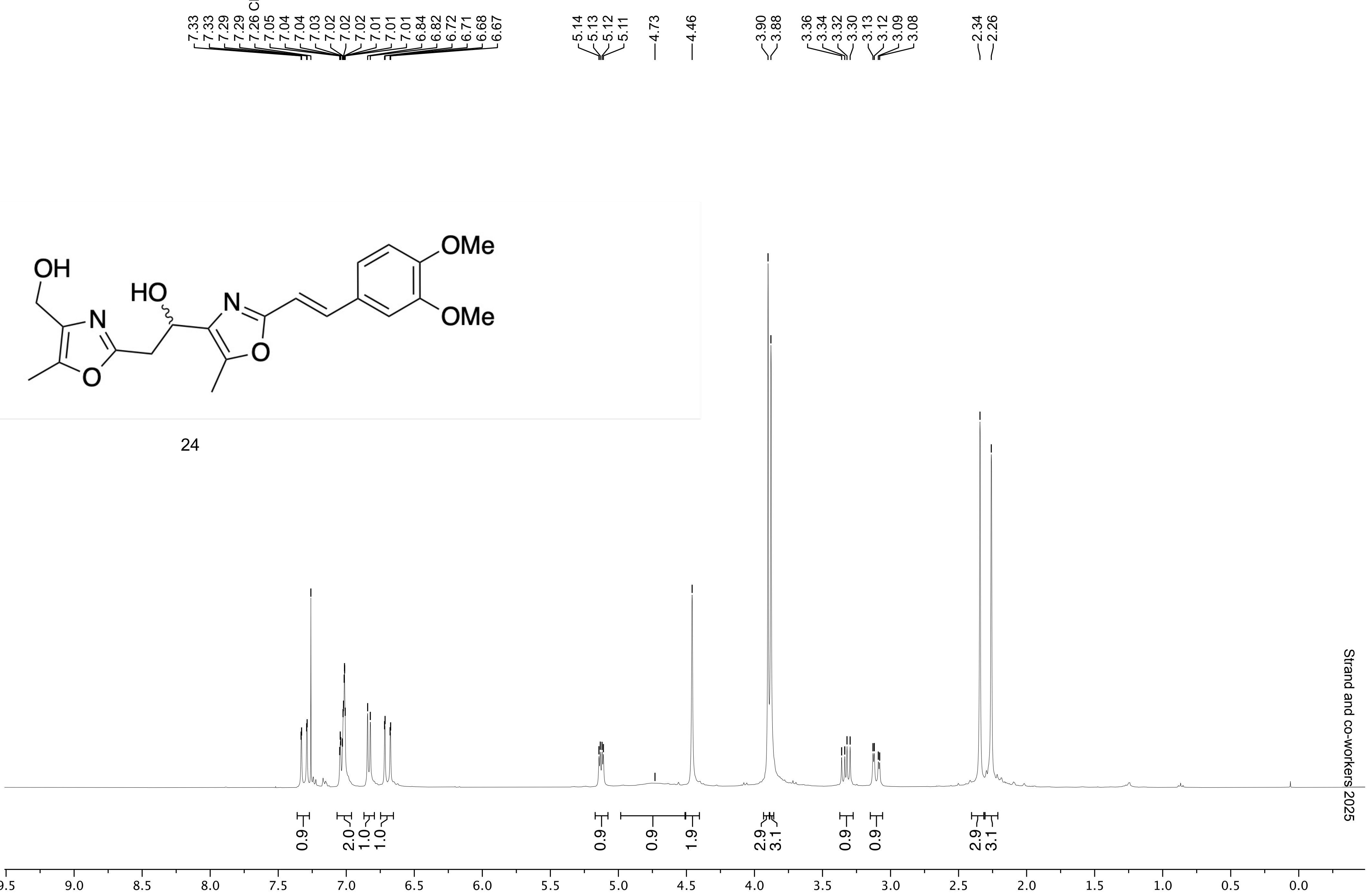

24

Strand and co-workers 2025

$^{13}C\{^1H\}$ NMR, $CDCl_3$, 101 MHz

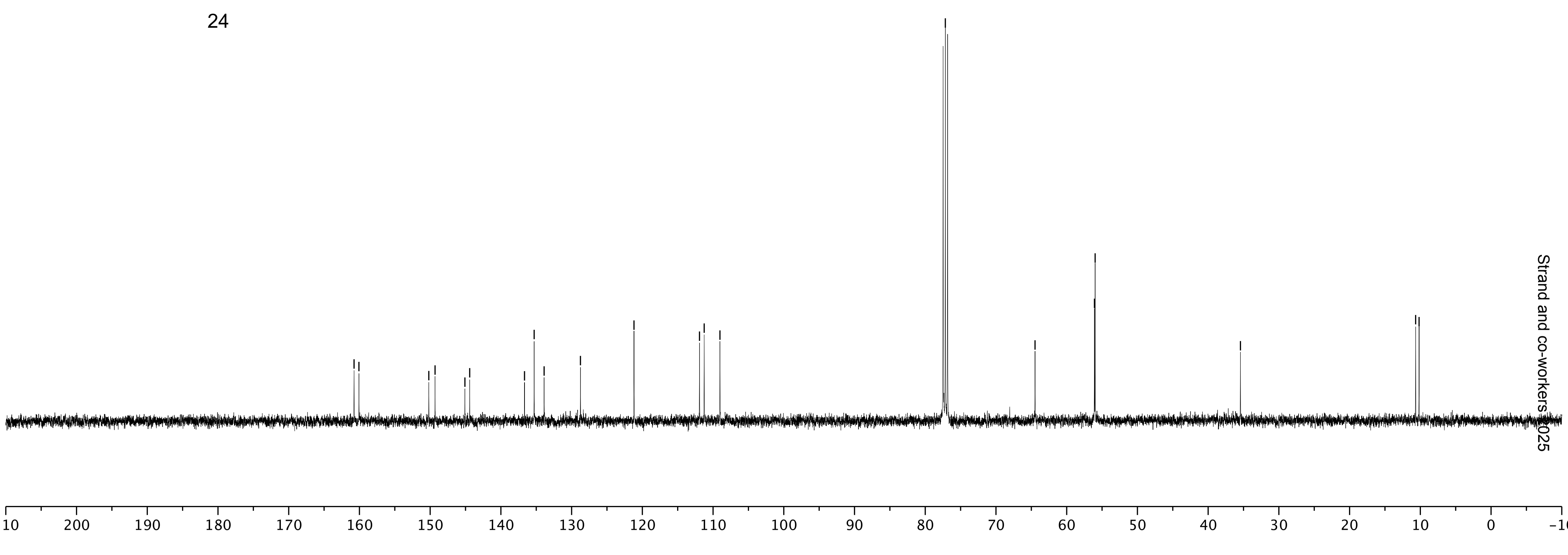

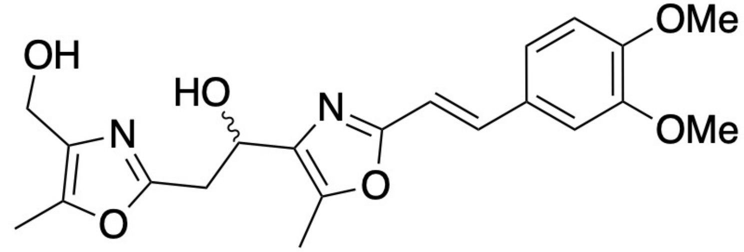

24

Strand and co-workers 2025

^{1}H NMR, $CDCl_3$, 400 MHz

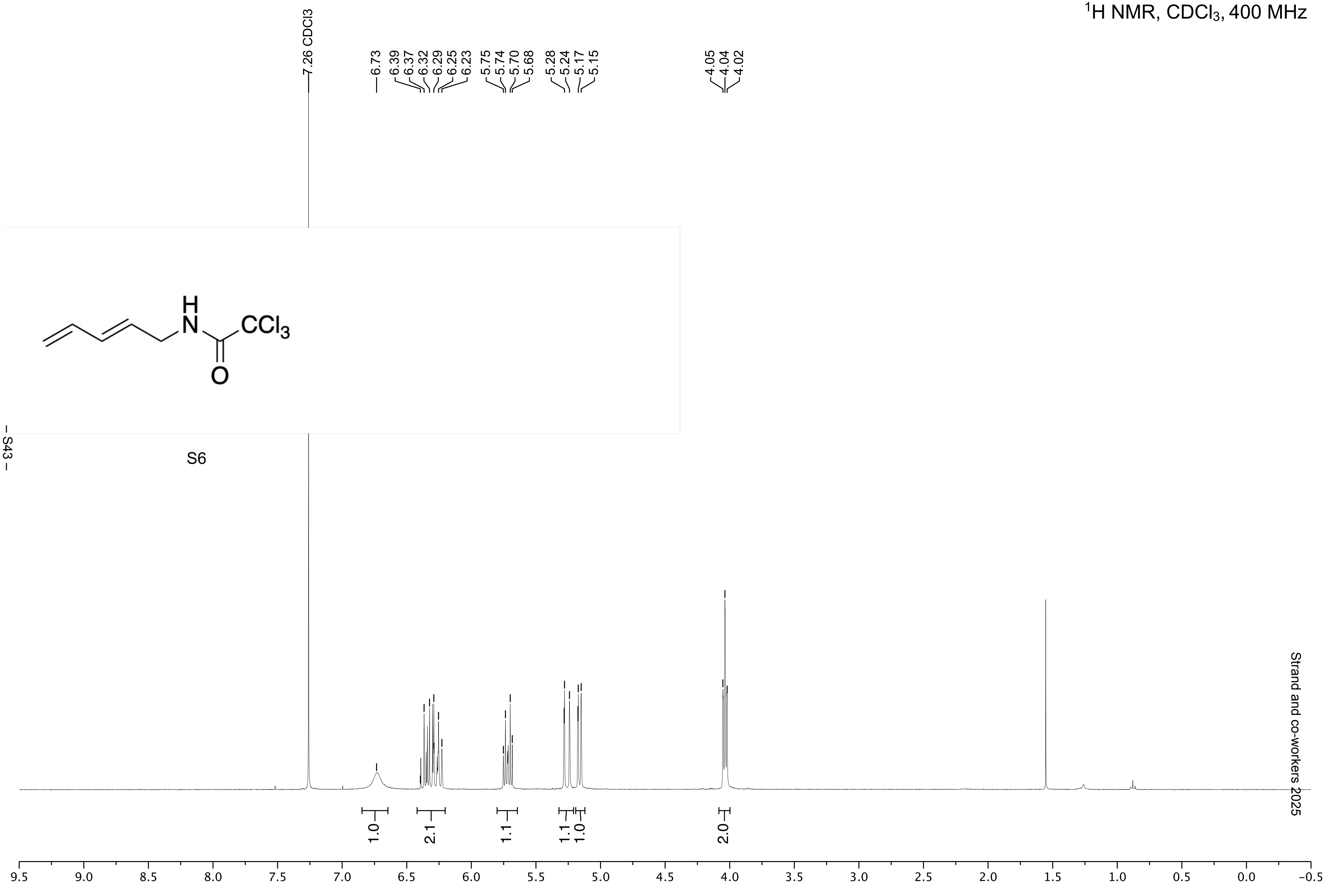

S6

Strand and co-workers 2025

^{1}H NMR, $CDCl_3$, 400 MHz

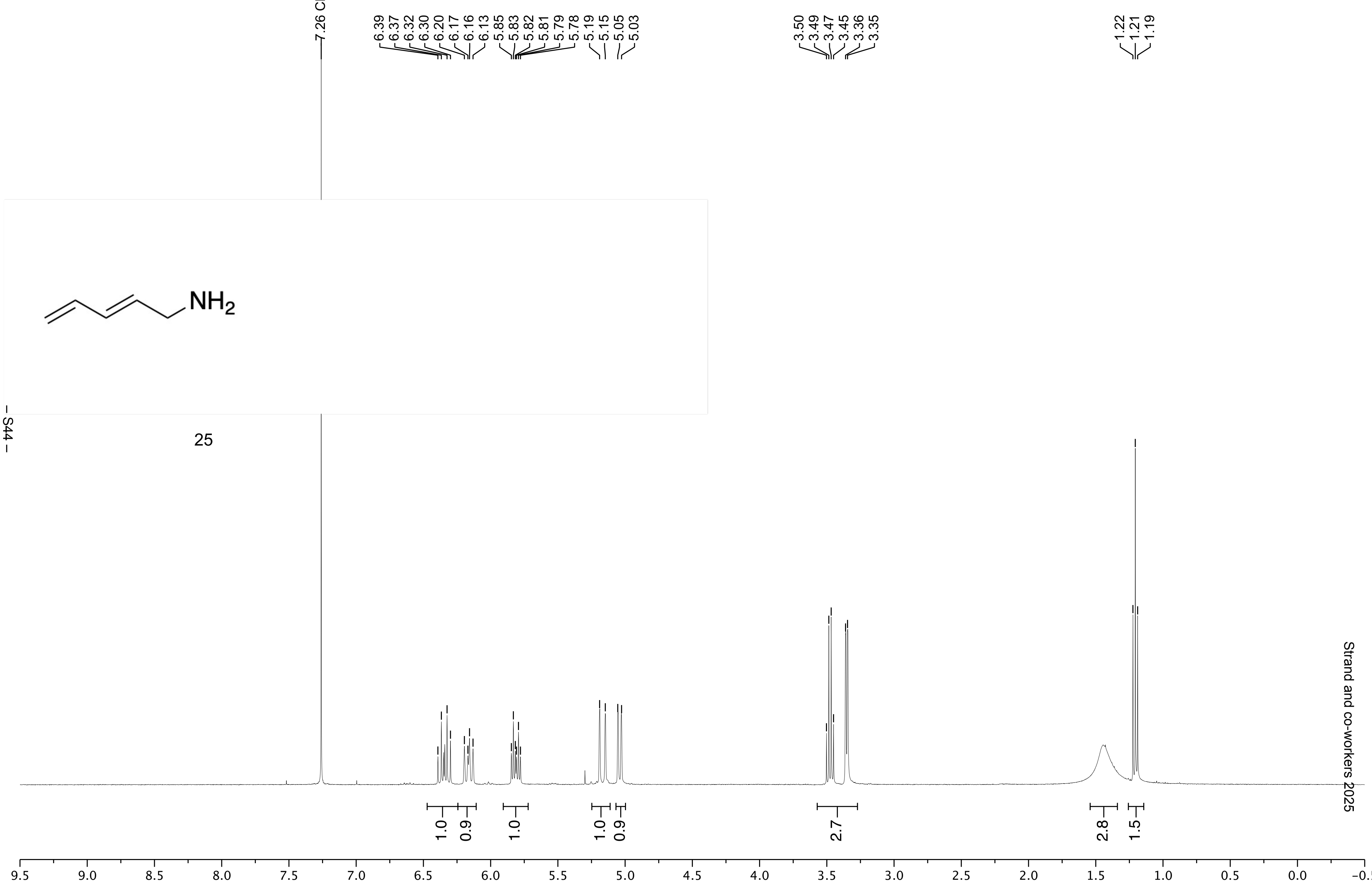

25

^1H NMR, C_6D_6, 400 MHz

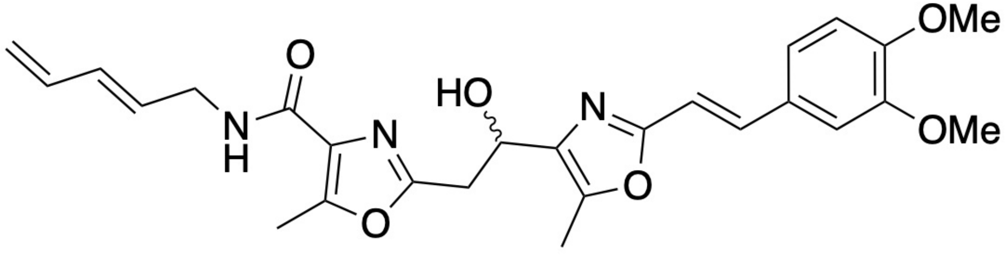

26a

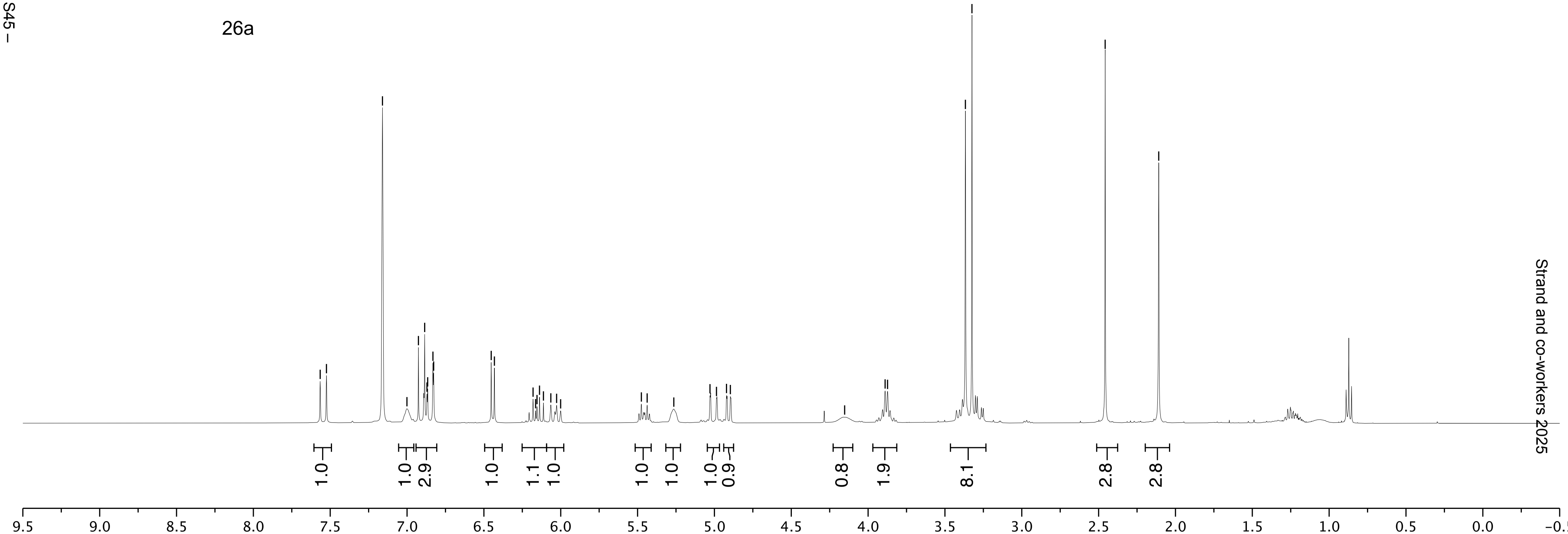

$^{13}C\{^{1}H\}$ NMR, $CDCl_3$, 101 MHz

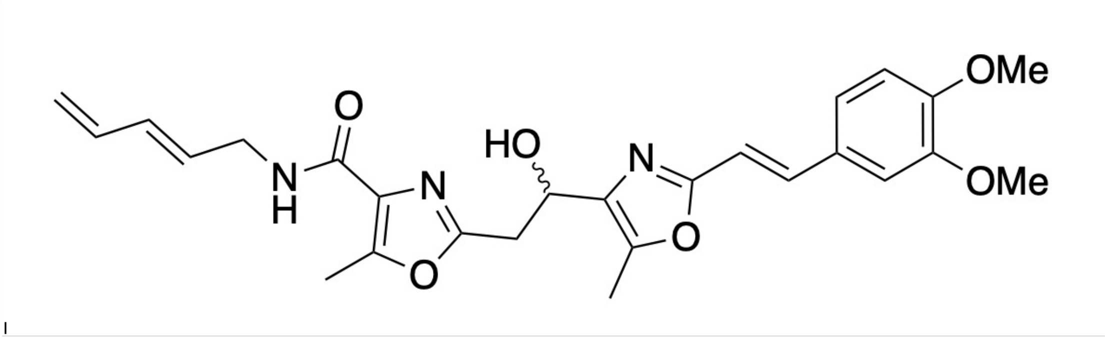

26a

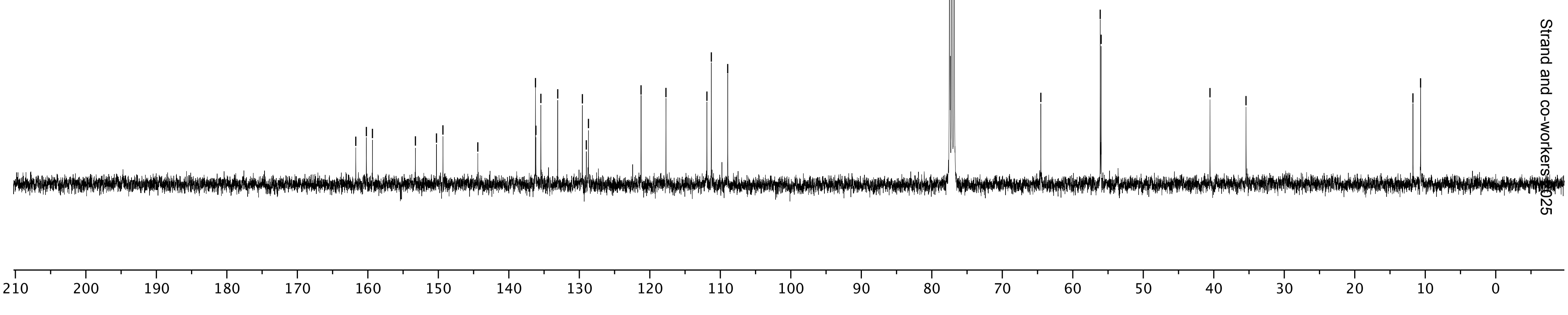

Strand and co-workers 2025

¹H NMR, $CDCl_3$, 400 MHz

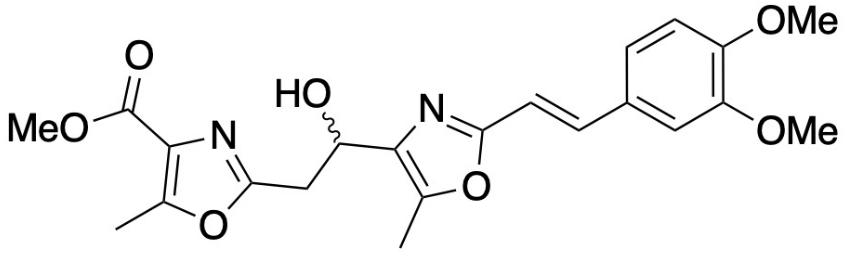

26b

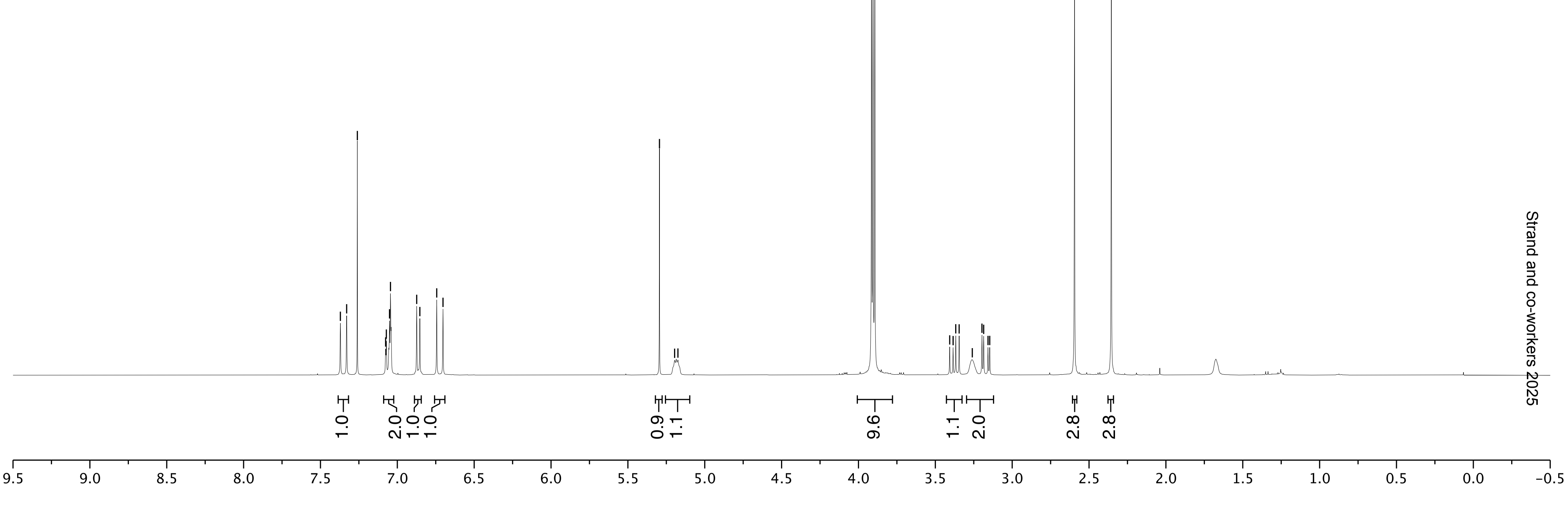

$^{13}C\{^1H\}$ NMR, $CDCl_3$, 101 MHz

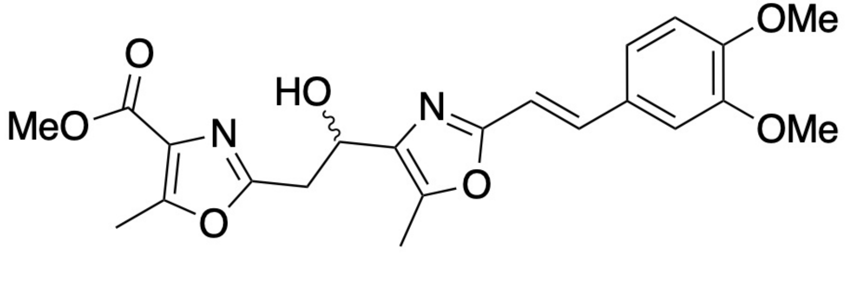

26b

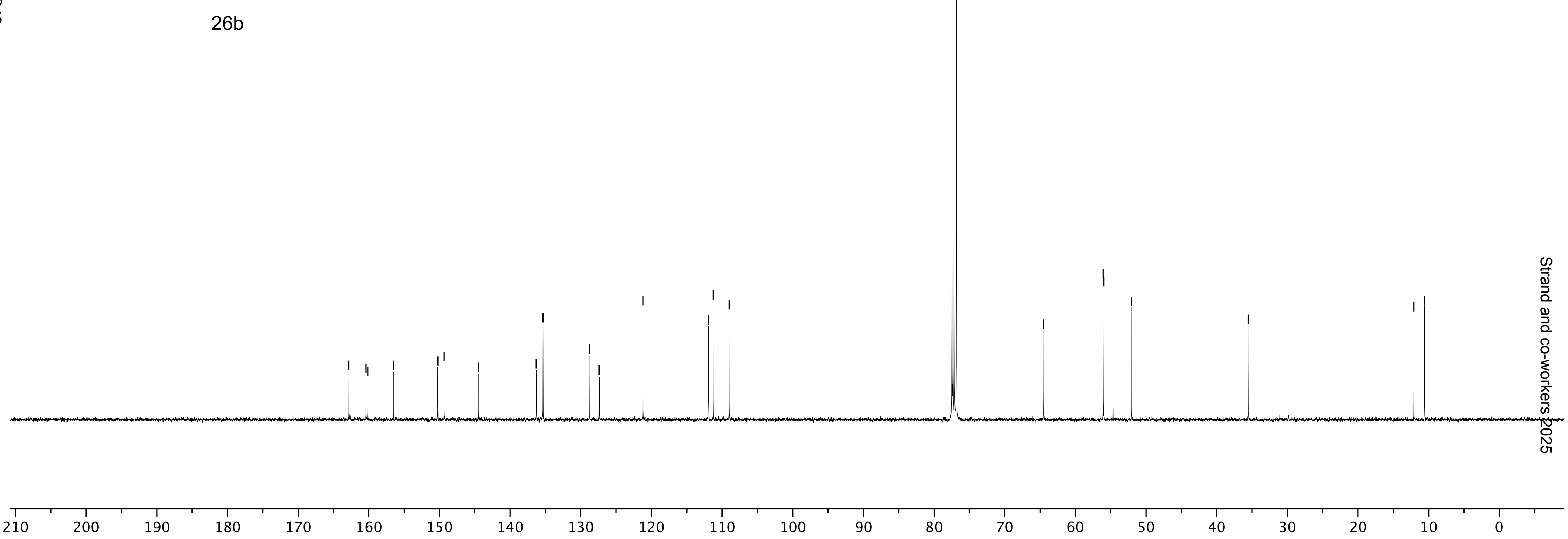

Strand and co-workers 2025

^{1}H NMR, acetone-d_6, 400 MHz

Strand and co-workers 2025

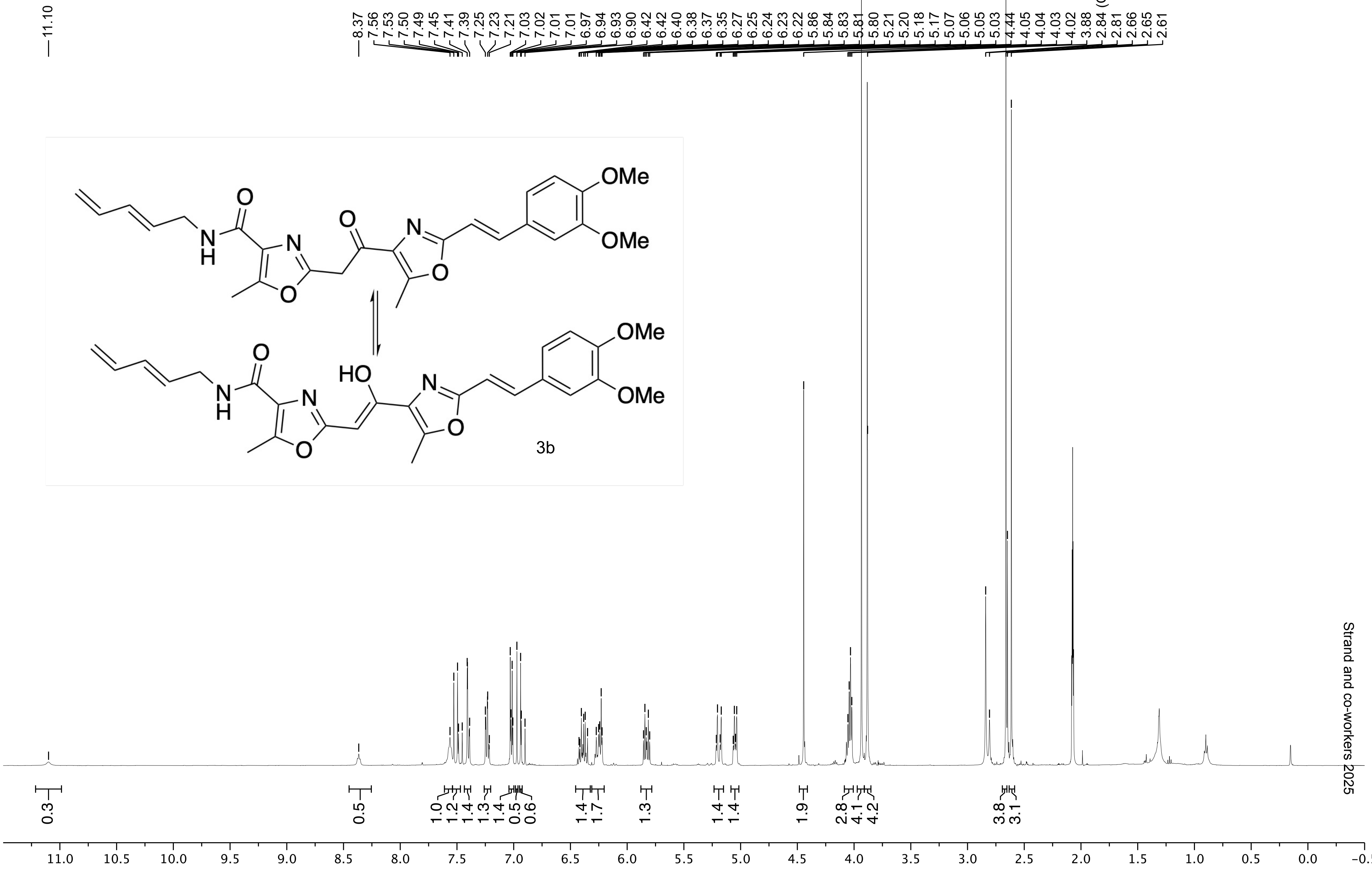

$^{13}C\{^{1}H\}$ NMR, acetone-d_6, 101 MHz

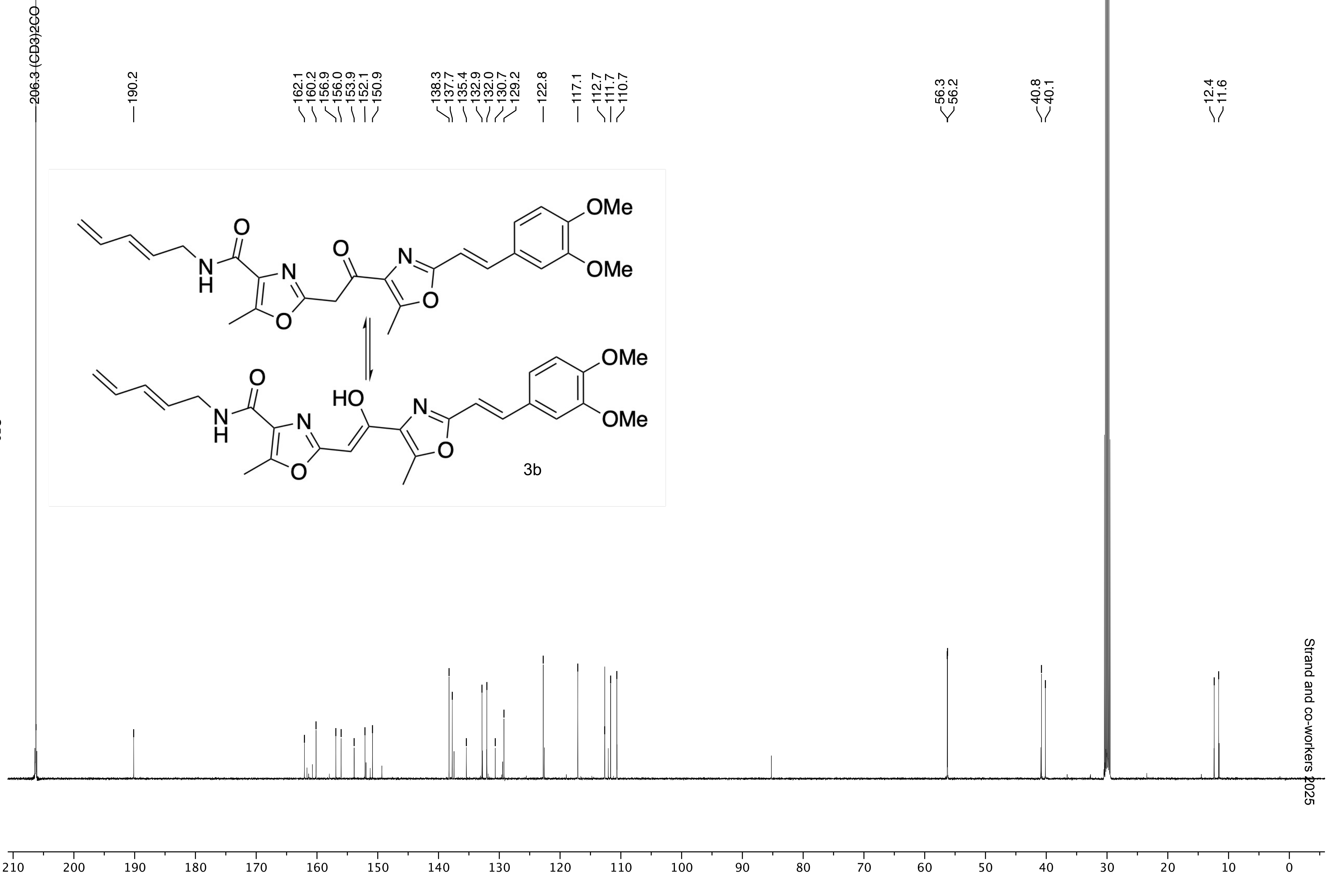

Supplement: Supplementary file 1 — Supporting Information [file CHEM-31-e202501394-s001.pdf]
